# Transitional care after hospitalization for sepsis in Germany– results from the population-based AVENIR cohort study

**DOI:** 10.1007/s15010-025-02589-7

**Published:** 2025-07-08

**Authors:** Thomas Ruhnke, Josephine Storch, Antje Freytag, Norman Rose, Aurelia Kimmig, Patrik Dröge, Lisa Wedekind, Christian Günster, Ludwig Goldhahn, Enno Swart, Mathias W. Pletz, Konrad Reinhart, Peter Schlattmann, Carolin Fleischmann-Struzek

**Affiliations:** 1 AOK Research Institute (WIdO), Rosenthaler Strasse 31, 10178 Berlin, Germany; 2 Institute of General Practice and Family Medicine, Jena University Hospital, Jena, Germany; 3 Institute of Infectious Diseases and Infection Control, Jena University Hospital, Jena, Germany; 4 Center for Sepsis Control and Care, Jena University Hospital, Jena, Germany; 5 Institute of Medical Statistics, Computer and Data Sciences, Jena University Hospital, Jena, Germany; 6 Institute of Social Medicine and Health Systems Research (ISMHSR), Otto- von-Guericke-University Magdeburg, Magdeburg, Germany; 7 Sepsis Foundation, Berlin, Germany

**Keywords:** Sepsis, Transitional care, Health care utilization, Sankey diagrams, Survivor

## Abstract

**Purpose:**

Sepsis survivorship is associated with significant long-term morbidity, mortality and health care utilization. Transitional care between inpatient and follow-up care is crucial, but insufficiently understood. We investigated health care utilization in sepsis survivors 90 days post-discharge, comparing translational care during 2016–2019 vs. 2020 in the first year of the pandemic.

**Methods:**

This retrospective cohort study used nationwide health claims data of the “AOK– die Gesundheitskasse”. Sepsis patients with inpatient treatment in 2016–2019 were identified using explicit ICD-10 codes for sepsis and codes for organ dysfunction. A second sepsis patient cohort was identified in 2020, which included also explicitly defined sepsis patients as well as patients with COVID-19 and Influenza with evidence of organ dysfunction. Among survivors, health care utilization in the 90 days post-discharge was assessed and first health service provider contacts were visualized using Sankey diagrams.

**Results:**

Among 234,874 sepsis survivors in 2016–2019, 94.4% were treated by a general practitioner, 47.7% had ≥ 1 hospital readmission and 42.8% of patients had ≥ 1 emergency treatment 90 days post-sepsis. Nearly all patients had prompt health service provider contacts in that time frame, with physicians in the outpatient sector being the most common first and second health service provider contacts. In the 2020 cohort (*n* = 69,432 survivors), more patients died without follow-up contact. Additionally, the latency to the first and second health service provider contacts were elevated compared to 2016–2019.

**Discussion:**

Sepsis survivors receive early, high-frequency follow-up care in the inpatient and outpatient sector. This may be an opportunity to implement early screening for sequelae and targeted therapies.

**Supplementary Information:**

The online version contains supplementary material available at 10.1007/s15010-025-02589-7.

## Introduction

Sepsis is the most severe complication from infectious diseases, in which a dysregulated host response leads to organ failure [1]. Although acute mortality is around 40% in Germany [2], a growing number of patients survive the acute disease. The majority of these survivors are affected by a significant long-term morbidity and mortality [3], which is summarized as Post-Sepsis-Syndrome (PSS) [4]. PSS can impede return to work [5], lead to a decreased health-related quality of life [6] and is associated with high health care costs [3]. Comprehensive follow-up care for sepsis survivors is therefore recommended in the international sepsis guidelines [7], and can contribute to frequent readmissions and long-term mortality [8]. With regard to structured aftercare, the 90 days after hospital discharge are a particularly important time frame, as patients experience the highest post-acute mortality and hospital readmissions during that period of time [3, 9]. Furthermore, screening for long-term cognitive, psychological and physical impairments is recommended in the 2–4 weeks after discharge for the first time [10], underlining the need for seamless transitions between health care sectors. However, to date, health care utilization behavior of patients in this time frame is not well understood [11].

In this study, we aimed to generate insights on transitional sepsis care in Germany using nationwide health claims data. We focused on four main research questions:

(i) Which health service providers are involved in transitional care of sepsis survivors in the first 90 days post-discharge?
(ii) Which are the first health service provider contacts, and when do they take place?
(iii) Do vulnerable patients, e.g. living in nursing home before the sepsis index event, after intensive care unit (ICU)-treated sepsis or the elderly, enter care via different pathways than patients who do not belong to these vulnerable groups?
(iv) Did the COVID-19 pandemic in 2020 affect the translational care of sepsis survivors after hospitalization?

## Methods

The study was pre-registered (DRKS00031302) and approved by the Jena University Hospital institutional review board (2023-2992-Daten).

### Data source

In this population-based observational cohort study, we used health claims data from eleven legally independent statutory health insurance funds of the ‘AOK– Die Gesundheitskasse’ (local health care funds) covering around one third of the German population. The AOK Research Institute (WIdO) provided data from 2015 to 2021. In Germany, statutory health insurance is the primary form of healthcare coverage. Enrolment to statutory health insurance is unrestricted regarding age, health status, income, employment, or geographical region. As part of routine healthcare administration, statutory health insurance funds collect data for billing purposes. These data include detailed information on both outpatient and inpatient treatments provided to insured individuals, such as diagnoses (ICD codes), procedures (OPS codes), treatment dates, and provider details. Since all hospitals in Germany are required to treat patients insured under the statutory health insurance, the inpatient data comprehensively cover all hospitals nationwide.

### Patients

The study population included patients aged ≥ 16 years with an inpatient hospitalization for sepsis between 2016 and 2020 identified by explicit ICD-10 codes for sepsis combined with ICD-10-codes or procedural (OPS) codes for organ dysfunction. ICD-10-GM codes were considered if they were coded as primary or secondary hospital discharge diagnoses. In Germany, ICD-10 codes for sepsis were defined according to the sepsis-1/2 definition for severe sepsis [12, 13] until the end of 2019. As coding was adapted to the new sepsis-3 definitions in 2020 [1], we omitted the code R65.1 as sepsis code and extended the definitions to cases with an ICD-10 code for Covid-19 (ICD-10-GM: U07.1!) or Influenza (ICD-10-GM: J09-J10) combined with a code indicating an acute organ dysfunction. The detailed selection criteria are described in the previously published study protocol [14]. The first hospitalization of a patient with sepsis during the observation period is referred to as the index case. The index case includes also interhospital-transfers from the index hospital and all consecutive hospitals if an admission to another hospital occurred within one day of discharge. The observation period of 90 days started with the discharge from the last hospital in which the patient was treated during the index case including interhospital-transfers.

**Fig. 1:**
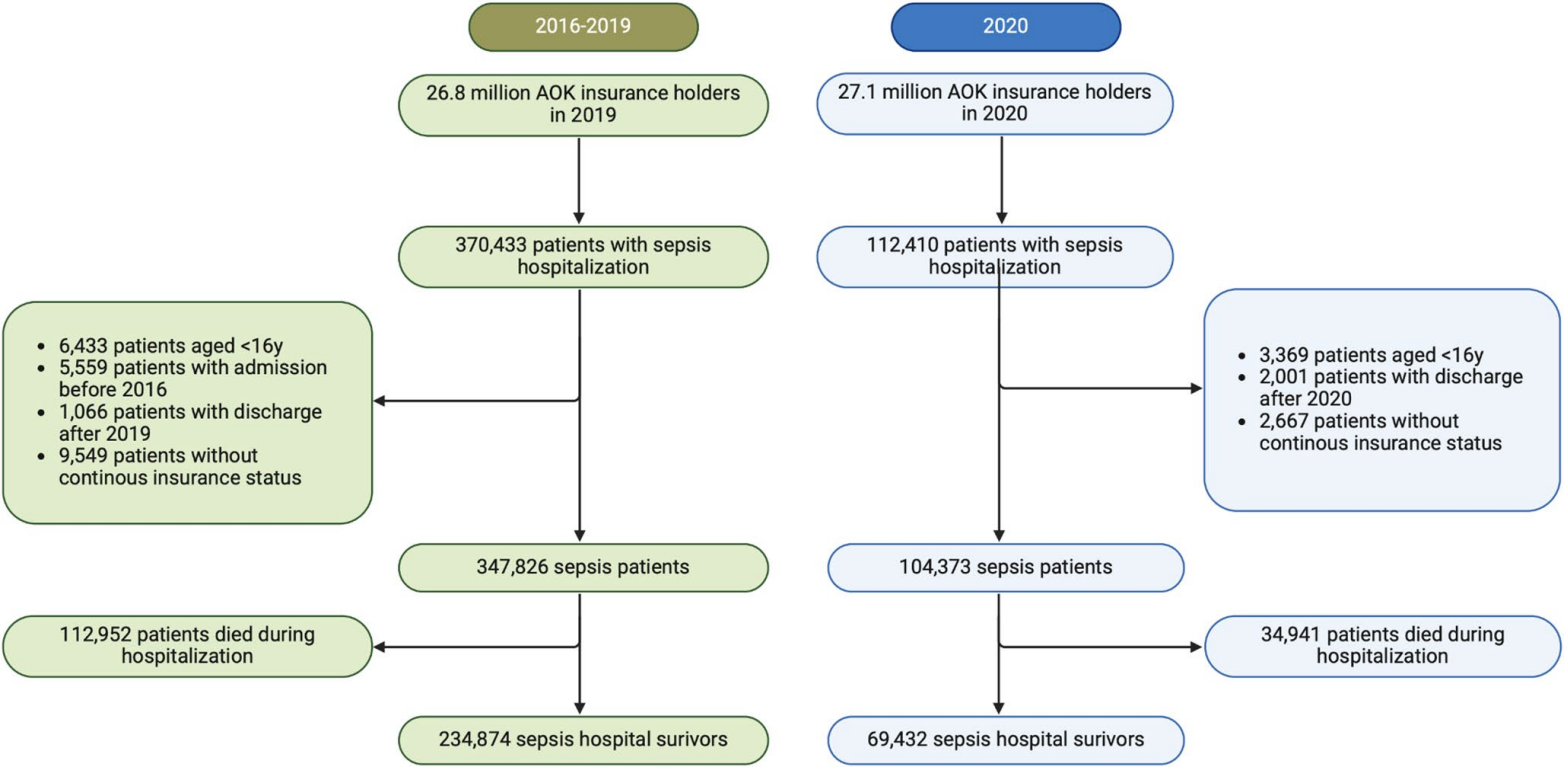
Flow of patient inclusion

Due to the change in sepsis coding and the COVID-19 pandemic, we analyzed two cohorts with index treatment in 2016–2019 and index treatment in 2020. We excluded patients with admission before 2016 and patients with discharge after 2019 (after 2020 for the 2020 cohort). Only patients with continuous insurance status 12 months before and 24 months (12 months for the 2020 cohort) after index treatment or until death are included in the study. Finally, we excluded all patients who died during the hospitalization (Fig. 1). We analyzed transitional care starting from the day of discharge from the last interhospital-transfer from the index treatment.

### Outcomes

We assessed patient health care utilization in the first 90 days after index hospitalization including outpatient general practitioner (GP) and specialist contacts (both planned and unplanned contacts), inpatient and outpatient hospital treatments, and inpatient rehabilitation. We also examined emergency treatment, specifically: treatment by outpatient emergency service of the association of statutory health insurance physicians, outpatient treatments in emergency departments in hospitals (without inpatient admission) and hospital stays with emergency admissions. For hospital and rehabilitation treatments, admission date was used for categorization. In the outpatient sector, we detected physician-patient contact exact to the day if a fee schedule position (Gebührenordnungsposition– GOP) was billed which indicated a personal or telemedical contact. Outpatient GPs and specialists were identified based on specific capitation fees they can bill once in a quarter when seeing the patients (see Table S2 in the Supplementary Material). For outpatient cases with multiple capitation fees, the physician group of the lifelong physician number was additionally used to distinguish between the groups.

Using Sankey diagrams [15], we focused on the first two physician-patient contacts after the index treatment. Sankey diagrams in general display flows between successive steps through arrows whose thickness is proportional to flow quantity without loss of information [15]. In our context, they were used to depict the distribution and transitions of patients through different levels of care (e.g., outpatient visits, hospital readmissions), allowing for an intuitive representation of post-sepsis healthcare trajectories. Index treatments without follow-up treatment were explicitly highlighted and an additional category was created for patients who died after the index treatment. If there were several physician-patient contacts on one day, then the contacts were assigned to an ‘other combinations’ category.

### Statistical analyses

We report proportions with 95% confidence intervals (CI), means with standard deviation (SD) and medians with interquartile ranges (IQR). We analyzed all outcomes for the total population of hospital survivors and the subgroups of age groups (< 40, 40–64, 65–79, ≥ 80), survivors with and without pre-existing nursing care dependency (nursing care level < 2, nursing care level ≥ 2 without nursing home residence, nursing home residence; nursing care levels entitle patients to long-term care insurance benefits and are defined as follows: level 1: “Minor impairment of independence”, level 2: “Substantial impairment of independence”, level 3: “Severe impairment” level 4: “Severest impairment”, level 5: “Severest impairment and special care requirements”) as well as ICU-treated and non-ICU-treated sepsis survivors (for definitions, see subgroup definitions in the Supplementary Material). All descriptive analyses were performed with Oracle 12c or rather R (version 4.4.1) and R Studio (version 2024.04.1) using the package gtsummary. Data preparation for Sankey diagrams was performed using Oracle 12c, and the Sankey diagrams were created with Python 3.10 using the package plotly 5.22.0.

## Results

We identified 347,826 sepsis patients between 2016 and 2019, of which 234,874 (67.5%) survived the hospitalization (Fig. 1). Mean age of survivors was 71.8 (SD 14.1) years, and 45.8% were female (Table 1). The most common focus of infection was the respiratory tract in 39.5% of cases; and 9.6% had an ICD-10-GM code for septic shock. 37.0% of survivors had received ICU complex treatment.

**Table 1:** Patient demographics and clinical features of the AVENIR cohort (2016–2019, 2020)

Variable	2016–2019		2020	
	*N* = 234,**874**	95% CI^1^	*N* = 69,**432**	95% CI^1^
**Demographics and comorbidity**				
Age, mean (SD); median (Q1, Q3)	71.8 (14.1); 75 (64; 82)		70.0 (15.4); 73 (60; 82)	
Sex, female, n (%)	107,474 (45.8%)	[45.6%, 46.0%]	32,317 (46.5%)	[46.2%, 46.9%]
Nursing care grade, n (%)				
0	128,951 (54.9%)	[54.7%, 55.1%]	39,122 (56.3%)	[56.0%, 56.7%]
1	4,284 (1.8%)	[1.6%, 2.0%]	2,328 (3.4%)	[3.0%, 3.7%]
2	37,783 (16.1%)	[15.9%, 16.3%]	10,183 (14.7%)	[14.3%, 15.0%]
3	32,050 (13.6%)	[13.4%, 13.9%]	9,678 (13.9%)	[13.6%, 14.3%]
4	21,650 (9.2%)	[9.0%, 9.4%]	5,862 (8.4%)	[8.1%, 8.8%]
5	10,156 (4.3%)	[4.1%, 4.5%]	2,259 (3.3%)	[2.9%, 3.6%]
Pre-existing nursing home residence, n (%)	30,210 (12.9%)	[12.7%, 13.0%]	8,341 (12.0%)	[11.8%, 12.3%]
Pre-exisiting long-term mechanical ventilation, n (%)	4,377 (1.9%)	[1.8%, 1.9%]	1,044 (1.5%)	[1.4%, 1.6%]
Pre-existing dialysis, n (%)	10,031 (4.3%)	[4.2%, 4.4%]	2,417 (3.5%)	[3.3%, 3.6%]
Charlson Index (unweighted), mean (SD), median (Q1, Q3)	3.6 (2.3); 3 (2; 5)		3.1 (2.3); 3 (1; 5)	
Employment status (employed), n (%)	35,247 (15.0%)	[14.9%, 15.2%]	14,256 (20.5%)	[20.2%, 20.8%]
**Acute sepsis**				
Septic shock, n(%)	22,464 (9.6%)	[9.4%, 9.7%]	5,512 (7.9%)	[7.7%, 8.1%]
Organ dysfunctions, n (%)				
Respiratory failure	119,480 (50.9%)	[50.7%, 51.1%]	42,553 (61.3%)	[60.9%, 61.6%]
Coagulopathy	39,343 (16.8%)	[16.6%, 16.9%]	9,301 (13.4%)	[13.1%, 13.7%]
Cardiovascular failure	99,267 (42.3%)	[42.1%, 42.5%]	28,059 (40.4%)	[40.0%, 40.8%]
Liver failure	3,884 (1.7%)	[1.6%, 1.7%]	1,137 (1.6%)	[1.5%, 1.7%]
Renal failure	59,057 (25.1%)	[25.0%, 25.3%]	15,889 (22.9%)	[22.6%, 23.2%]
Encephalopathy	57,644 (24.5%)	[24.4%, 24.7%]	13,860 (20.0%)	[19.7%, 20.3%]
Number of organ dysfunctions (mean (SD), median (Q1, Q3))	1.6 (1.0); 1 (1; 2)		1.6 (1.0); 1 (1; 2)	
Focus of infection, n (%)				
Lower respiratory tract infection	92,664 (39.5%)	[39.3%, 39.7%]	35,068 (50.5%)	[50.1%, 50.9%]
Urinary system	87,540 (37.3%)	[37.1%, 37.5%]	20,414 (29.4%)	[29.1%, 29.7%]
Skin, wound, soft tissue	25,993 (11.1%)	[10.9%, 11.2%]	5,451 (7.9%)	[7.7%, 8.1%]
Intraabdominal, retroperitoneal	27,233 (11.6%)	[11.5%, 11.7%]	6,145 (8.9%)	[8.6%, 9.1%]
Intrathoracic	3,874 (1.6%)	[1.6%, 1.7%]	901 (1.3%)	[1.2%, 1.4%]
CNS	2,733 (1.2%)	[1.1%, 1.2%]	568 (0.8%)	[0.8%, 0.9%]
Bloodstream, vascular infections	19,256 (8.2%)	[8.1%, 8.3%]	3,804 (5.5%)	[5.3%, 5.7%]
Bone, joints	7,512 (3.2%)	[3.1%, 3.3%]	1,569 (2.3%)	[2.2%, 2.4%]
GI-tract, diarrhea	27,035 (11.5%)	[11.4%, 11.6%]	6,733 (9.7%)	[9.5%, 9.9%]
Systemic viral infections	3,933 (1.7%)	[1.6%, 1.7%]	26,046 (37.5%)	[37.2%, 37.9%]
Genital organs, sexually transmitted infections	4,376 (1.9%)	[1.8%, 1.9%]	941 (1.4%)	[1.3%, 1.4%]
Unspecific infections	140,999 (60.0%)	[59.8%, 60.2%]	37,657 (54.2%)	[53.9%, 54.6%]
Devise-related infections	18,404 (7.8%)	[7.7%, 7.9%]	3,447 (5.0%)	[4.8%, 5.1%]
Infection with seasonal Influenza	2,315 (1.0%)	[0.9%, 1.0%]	4,566 (6.6%)	[6.4%, 6.8%]
Infection with SARS-CoV-2	-	-	25,226 (36.3%)	[36.0%, 36.7%]
Nosocomial infection, n (%)	71,388 (30.4%)	[30.2%, 30.6%]	15,662 (22.6%)	[22.2%, 22.9%]
**Clinical features**				
Mechanical ventilation, n (%)	53,704 (22.9%)	[22.7%, 23.0%]	13,133 (18.9%)	[18.6%, 19.2%]
Renal replacement therapy, n (%)	17,846 (7.6%)	[7.5%, 7.7%]	4,257 (6.1%)	[6.0%, 6.3%]
Hospital length of stay (mean (SD); median (Q1, Q3))	22.8 (22.0); 16 (9; 29)		19.6 (20.3); 13 (8; 25)	
ICU treatment, n (%)	86,996 (37.0%)	[36.8%, 37.2%]	21,515 (31.0%)	[30.6%, 31.3%]
Surgical treatment, n (%)	93,513 (39.8%)	[39.6%, 40.0%]	20,301 (29.2%)	[28.9%, 29.6%]

^1^ CI = Confidence Interval

### Health service utilization in the first 90 days post discharge in 2016–2019

In the 90 days post-sepsis, 94.4% and 58.8% of patients received GP and specialist treatment in the outpatient setting, respectively (Table 2). Urologists, psychiatrists and ENT specialists were the most common specialists involved in patient treatment (16.3%, 8.0% and 7.6% of patients with any contact, respectively). 47.7% had at least one hospital readmission. In sum, 42.8% of patients had any kind of emergency treatment 90 days post-sepsis. A total of 32.6% had a hospital stay with emergency admission, 12.8% were treated by the outpatient emergency service of the statutory health insurance physicians and 10.3% of patients received outpatient treatment in an emergency department (without inpatient admission). Health care utilization in the subgroups is shown in Supplementary Material Tables S3 (by age group), S4 (by pre-existing nursing care dependency) and S5 (by ICU complex treatment).

**Table 2:** Health care utilization in the 90 days post discharge

Outcome	2016–2019		2020	
	*N* = 234,**874**	95% CI^1^	*N* = 69,**432**	95% CI^1^
At least one outpatient physician contact, n (%)	226,830 (96.6%)	[96.5%; 96.6%]	66,095 (95.2%)	[95.0%; 95.4%]
Number of outpatient physician contacts, mean (SD); median (Q1; Q3)	6.9 (5.7); 6 (3; 9)		6.2 (5.5); 5 (3; 8)	
At least one outpatient general practitioner contact, n (%)	221,605 (94.4%)	[94.3%; 94.4%]	64,151 (92.4%)	[92.2%; 92.6%]
Number of outpatient general practitioner contact, mean (SD); median (Q1; Q3)	5.1 (4.5); 4 (2; 7)		4.5 (4.2); 4 (2; 6)	
At least one outpatient specialist contact, n (%)	138,132 (58.8%)	[58.6%; 59.0%]	39,852 (57.4%)	[57.0%; 57.8%]
Number of outpatient specialist contact, mean (SD); median (Q1; Q3), in detail:	1.8 (3.4); 1 (0; 2)		1.7 (3.4); 1 (0; 2)	
… ENT specialist	17,929 (7.6%)	[7.5%; 7.7%]	5,362 (7.7%)	[7.5%; 7.9%]
… cardiologist	13,870 (5.9%)	[5.8%; 6.0%]	4,749 (6.8%)	[6.7%; 7.0%]
… nephrologist	15,484 (6.6%)	[6.5%; 6.7%]	3,700 (5.3%)	[5.2%; 5.5%]
… neurologist	10,893 (4.6%)	[4.6%; 4.7%]	3,091 (4.5%)	[4.3%; 4.6%]
… pneumologist	9,450 (4.0%)	[3.9%; 4.1%]	4,354 (6.3%)	[6.1%; 6.5%]
… psychiatrist	18,790 (8.0%)	[7.9%; 8.1%]	5,513 (7.9%)	[7.7%; 8.1%]
… psychologist	835 (0.36%)	[0.33%; 0.38%]	385 (0.55%)	[0.50%; 0.61%]
… physician with specialization in psychosomatics	92 (0.04%)	[0.03%; 0.05%]	36 (0.05%)	[0.03%; 0.07%]
… pain treatment, pain therapy	1,840 (0.78%)	[0.75%; 0.82%]	545 (0.78%)	[0.72%; 0.85%]
… urologist	38,219 (16.3%)	[16.1%; 16.4%]	9,530 (13.7%)	[13.5%; 14.0%]
At least one inpatient hospital admission, n (%)	112,047 (47.7%)	[47.5%; 47.9%]	25,307 (36.4%)	[36.1%; 36.8%]
Number of inpatient hospital admissions, mean (SD); median (Q1; Q3)	0.8 (1.1); 0 (0; 1)		0.6 (1.0); 0 (0; 1)	
At least one outpatient hospital treatment, n (%)	23,931 (10.2%)	[10.1%; 10.3%]	6,584 (9.5%)	[9.3%; 9.7%]
Number of outpatient hospital treatments, mean (SD); median (Q1; Q3)	0.1 (0.5); 0 (0; 0)		0.1 (0.5); 0 (0; 0)	
At least one inpatient rehabilitation, n (%)	28,721 (12.2%)	[12.1%; 12.4%]	6,042 (8.7%)	[8.5%; 8.9%]
Number of inpatient rehabilitations, mean (SD); median (Q1; Q3)	0.1 (0.4); 0 (0; 0)		0.1 (0.3); 0 (0; 0)	
At least one emergency treatment, n (%)	100,448 (42.8%)	[42.6%; 43.0%]	23,881 (34.4%)	[34.0%; 34.7%]
Number of emergency treatments, mean (SD); median (Q1; Q3)	0.8 (1.2); 0 (0; 1)		0.6 (1.1); 0 (0; 1)	
At least one treatment by outpatient emergency service of the statutory health insurance physicians, n (%)	30,140 (12.8%)	[12.7%; 13.0%]	6,442 (9.3%)	[9.1%; 9.5%]
At least one treatment in emergency departments in hospitals (without inpatient admission), n (%)	24,116 (10.3%)	[10.1%; 10.4%]	6,199 (8.9%)	[8.7%; 9.1%]
At least one hospital stay with emergency admission, n (%)	76,659 (32.6%)	[32.4%; 32.8%]	17,663 (25.4%)	[25.1%; 25.8%]

^1^ CI = Confidence Interval

### First health service provider contacts for index treatment in 2016–2019

**Fig. 2:**
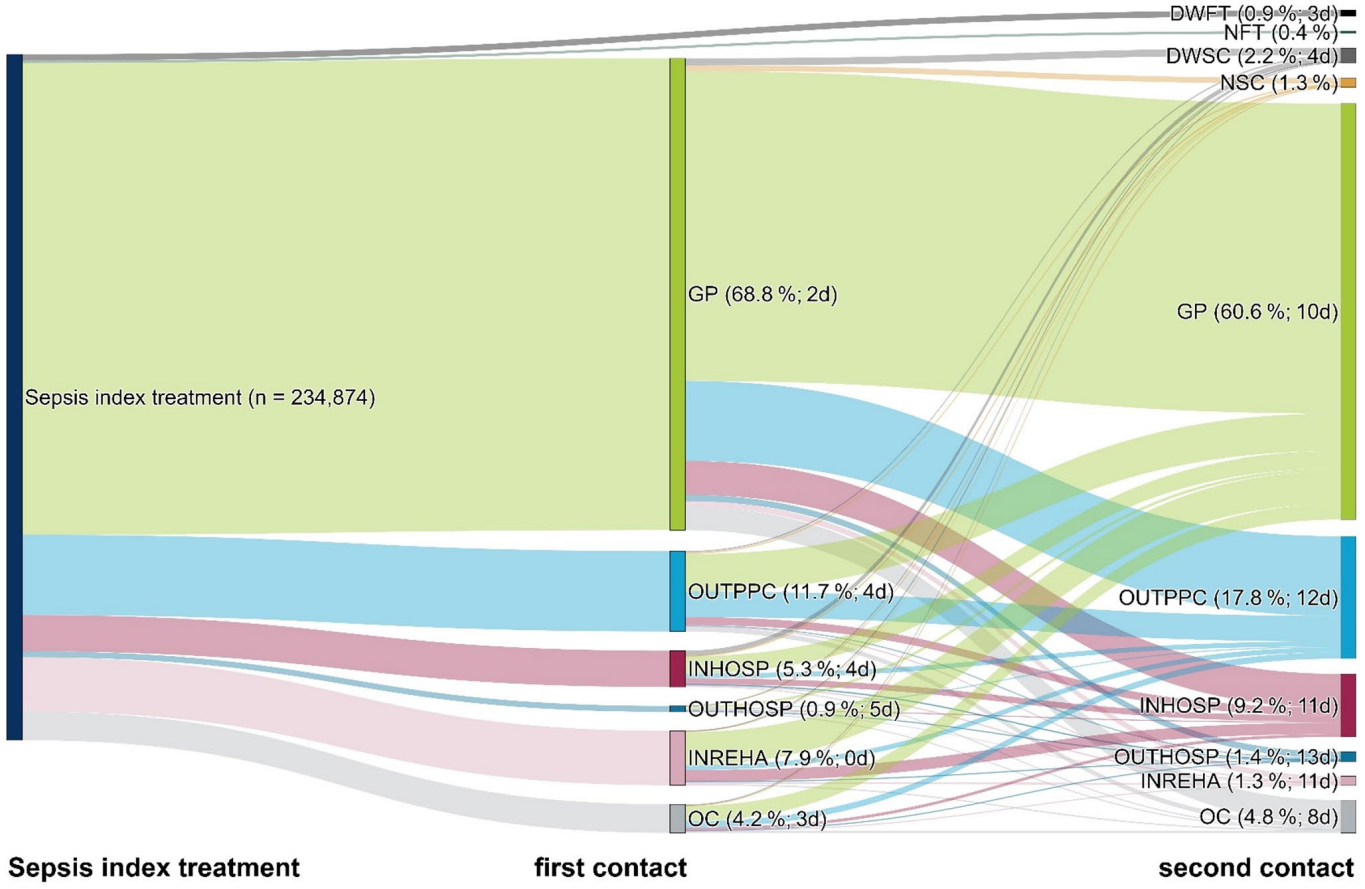
First health service provider contacts in the 90 days post-discharge 2016–2019 among *n* = 234,874 sepsis survivors. Legend for first and second contact: Category (proportion; Median number of days between health service provider contact and discharge from index hospitalization). Abbreviations: DWFT = died without follow-up treatment, DWSC = died without second contact, GP = general practitioner, INHOSP = inpatient hospital admissions (including emergency admissions), INREHA = inpatient rehabilitation; NFT = no further treatment, NSC = no second contact, OC = other combinations, OUTHOSP = outpatient hospital treatment, OUTPPC = other outpatient physician-patient contact (specialists, outpatient emergency treatments)

The Sankey diagram in Fig. 2 illustrates the pathways of patients’ first and second health care contact within 90 days after discharge from index hospitalization between 2016 and 2019. The width of each flow represents the proportion of patients following that specific pathway. Each contact is labeled with the type of provider, the proportion of patients involved, and the median number of days between discharge and contact. Within 90 days after the index treatment, 68.8% of survivors had consulted first a GP with a time latency of a median of 2 days, while 11.7% had their first contact with other outpatient physicians (e.g. specialists) in a median of 4 days in median (Fig. 2). First health service provider contacts in a rehabilitation facility or a hospital were less common and occurred in 7.9% and 5.3% of cases, respectively. There was heterogeneity in second contacts. The GP was also most frequently the second contact for 60.6% of cases with a median latency of 10 days after discharge, followed by other outpatient physicians in 17.8% of cases after a median of 12 days post-discharge. Only 0.9% of survivors died without any kind of further treatment, and another 0.4% of sepsis survivors had no contact with a physician within 90 days of the index event. First and second health service provider contacts occurred with only a short latency of between 0 and 5 days and 8–13 days after the discharge from the index hospitalization, respectively (Fig. 2).

In the subgroup analyses, we found that first contacts with GPs were least frequent in the < 40 age group (58.0%) and increased with age (40–64 years: 65.0%, 65–79 years: 67.1%, ≥ 80 years: 74.3%), while on the other hand first consultations with other outpatient physicians were more common among younger patients (17.4% (< 40 years) vs. 10.3% (≥ 80 years); see Supplementary Material Fig. S1a-S1d). Patients with ICU treatment had a higher proportion of first health service provider contacts in rehabilitation facilities (15.0% with vs. 3.8% without ICU treatment) and less first contacts in the outpatient sector (GPs: 64.9% vs. 71.1%, other outpatient physicians: 9.4% vs. 13.1%, respectively, see Supplementary Material Fig. S3a and S3b). Among patients with nursing care dependency, we found that patients with care level ≥ 2 or patients living in a nursing home showed higher rates of initial contacts with GPs (71.0% (care level ≥ 2), 74.0% (nursing home) vs. 66.4% (no nursing care dependency)) and lower rates of a first contact with a rehabilitation facility (4.8% (care level ≥ 2), 0.7% (nursing home) vs. 11.3% (no nursing care dependency)) (see Supplementary Material Fig. S2a - S2c).

### Changes in transitional care during the Covid-19 pandemic for index treatment in 2020

**Fig. 3:**
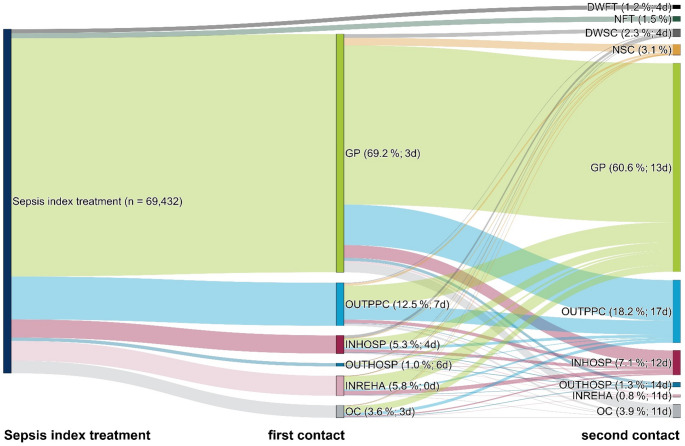
First health service provider contacts in the 90 days post-discharge 2020 among *n* = 69,432 sepsis survivors. Legend for first and second contact: Category (proportion; Median number of days between health service provider contact and discharge from index hospitalization). Abbreviations: DWFT = died without follow-up treatment, DWSC = died without second contact, GP = general practitioner, INHOSP = inpatient hospital admissions (including emergency admissions), INREHA = inpatient rehabilitation; NFT = no further treatment, NSC = no second contact, OC = other combinations, OUTHOSP = outpatient hospital treatment, OUTPPC = other outpatient physician-patient contact (specialists, outpatient emergency treatments

In 2020, our cohort consisted of 104,373 patients and 69,432 survivors (66.5% of the cohort vs. 67.5% in the 2016–2019 cohort). Mean age was lower than in 2016–2019 (70.0 (SD 15.4) years vs. 71.8 years) and more patients were female (46.5% vs. 45.8%, Table 1). Septic shock and ICU-treatment occurred less frequently (7.9% vs. 9.6% and 31.0% vs. 37.0%, respectively). A total of 36.3% of survivors had Covid-19-related sepsis and lower respiratory tract infection occurred more frequently (50.5% vs. 39.5%). Health care utilization 90 days post-sepsis was mostly decreased compared to 2016–2019 (Table 2). In the 90 days post-discharge, we found similar pattern of first health service provider contacts (Fig. 3) with two exceptions: Less patients had their first or second health care contact in rehabilitation facilities (5.8% and 0.8%, respectively, in 2020 vs. 7.9% and 1.3%, respectively, in 2016–2019), and a higher proportion of patients had no contact (1.5% vs. 0.4%) or died without further health care contacts (1.2% vs. 0.9%). The latency for the first and second patient-provider contact was higher in nearly all categories and ranged between 0 and 7 days and 11–17 days, respectively.

## Discussion

In this analysis of transitional care in sepsis patients, we found that sepsis survivors had a high frequent, multisector health care utilization in the 90 days post-sepsis. More than one third of survivors had at least one emergency treatment during that period. As common first health care provider contact, the GP plays a particular role in Germany, especially in patients of higher age, with chronic care needs and those who were treated outside the ICU setting. Of note, first health service provider contacts occurred with low latency of 0–7 days post-sepsis in median and nearly all patients had health care contacts in the quarter post-sepsis. This latency and the proportion of patients who died without further health care contacts post-sepsis, however, was considerably elevated during the first wave of the Covid-19 pandemic.

Our results are in line with previous research that confirms frequent post-acute readmissions in sepsis survivors [9]. According to a recent meta-analysis, 90-day readmission rate was 38.1% in sepsis survivors [9] and higher than in any other acute diseases [16], which may reflect a prolonged recovery period due to the severity of the acute disease, as well as chronic long-term impairments which can arise from lasting alterations in immune function and organ damage caused by the acute sepsis [17, 18]. In a matched US-cohort study, 42.6% of sepsis survivors were hospitalized in the 90 days post-sepsis, with a higher proportion of outpatient-care sensitive conditions (ACSC) than in patients with other acute medical diagnosis (42% vs. 37% of all readmissions) [16]. These ACSC hospitalizations are considered potentially preventable hospitalizations by early outpatient care [19], which underlines the opportunity to prevent hospital readmissions by seamless hospital follow-up for this vulnerable patient group.

To this end, our data confirm that nearly all survivors had early health care contacts in the 90 days post-discharge. More than two thirds of sepsis survivors were first treated by their GP, and more than 80% were first treated by a GP or other outpatient physician after their hospitalization, which therefore can be considered important stakeholders in transitional sepsis care with the potential to facilitate screening and coordinate treatment of post-sepsis sequelae. Nevertheless, in Germany, the structure of post-sepsis care remains highly heterogeneous, with no unified national guidelines or standardized care pathways, leading to considerable variability in follow-up practices and access to support services. Patients still subjectively report a lack of follow-up care, particularly with regard to coordination and sepsis-specific orientation in Germany [20]. This may be due to hurdles that currently hinder the provision of aftercare tailored to the needs of sepsis survivors in this context, including the lack of guidelines for specific aftercare after sepsis [11], the suboptimal information sharing between ICU and GPs, and capacity strains, e.g. in the availability of outpatient physical therapy [21]. Furthermore, GPs describe a scarcity of experience and knowledge on the aftercare of sequelae after critical illness due to their low incidence in the primary care context [21, 22], which underpins the potential of education on post-acute sepsis care for health care providers as a prerequisite for an improved aftercare [11]. The transition may also be facilitated by structured programs initiated during the acute hospitalization. In this regard, a recent randomized-controlled trial found that a nurse navigator program which started during the hospital stay and continued after discharge to initiate a review of the patients’ medication, the evaluation for new impairments or symptoms, the monitoring comorbidities, and palliative care approach when appropriate was effective in reducing a combined endpoint of 30-day readmissions and mortality [8].

Our study also marks differences in the provision of transitional care pre- and during the COVID-19 pandemic. Notably, the proportion of patients without post-sepsis health care contacts was elevated, the frequency of post-acute rehabilitation decreased, and the latency of first health service provider contacts increased in 2020 compared to 2016–2019. Similar observations were made for other acute and post-acute diseases in Germany, e.g. with longer durations between symptom onset and hospital treatment for acute cardiovascular diseases [23] or a lower number of rehabilitation treatments after stroke [24]. In the future, it seems therefore important to develop ways to keep the routine care of patients with acute illnesses requiring urgent care stable to prevent avoidable deaths and other adverse outcomes in the event of further public health crises.

This study has several strengths, including the comprehensive, population-based database with reliable data on health service provider contacts including more than 27 million insurance holders of the AOK in Germany. Precise operationalizations were developed to record health care utilization and outpatient contacts as comprehensively as possible. In particular, the operationalizations of physician-patient contacts via fee schedule positions and treatments in emergency departments provide novel insights compared to previous works. Another strength is that the study is that it is not subject to selection and healthy volunteer bias, as data from all AOK-beneficiaries could be included. This form of bias is often relevant in patients receiving life-threatening or intensive medical care, as their ability to give consent may be impaired.

However, there are also limitations to consider. We used nationwide health claims which were originally generated and collected for billing purposes. Previous validation studies showed an underestimation of sepsis cases in these data, as not all cases are diagnosed and coded as such, particularly if the sepsis severity is low [25]. Due to the billing processes in the outpatient sector, which occur on a quarterly basis, outpatient physician-patient contacts are likely underestimated because not all treatments can be billed separately, partly depending on the patient’s morbidity. This must be taken into account when interpreting the results. Furthermore, we cannot draw any conclusions about individual patient pathways and treatment decisions. We do not expect substantial differences in post-acute sepsis care between individuals insured by the AOK and those covered by other statutory health insurance providers in Germany, as the overall structure and access to services within the statutory health insurance system are largely comparable across health insurance providers. However, we cannot rule out demographic or socioeconomic differences between AOK-insured and non-AOK-insured populations [26], which may limit the generalizability of our findings to some extent. Moreover, it has to be noted that the 2016–2019 and 2020 cohorts are not directly comparable due to changes in coding guidelines in Germany and the incorporation of COVID-19 related sepsis cases in the 2020 cohort.

## Conclusions

After discharge, sepsis survivors receive early and high-frequency follow-up care in the inpatient and outpatient sectors in Germany, with the GP playing a prominent role in early care. This may be an opportunity to implement structured transition programs, and early screening for sequelae and targeted therapies, especially to avoid ACSC rehospitalizations as well as reduce excess mortality and morbidity in this population.

## Electronic supplementary material

Below is the link to the electronic supplementary material.

Supplementary Material 1

## Data Availability

The authors confirm that the data utilized in this study cannot be made available in the manuscript, the supplemental files, or in a public repository due to German data protection laws (‘Bundesdatenschutzgesetz’, BDSG). Therefore, they are stored on a secure drive in the WIdO, to facilitate replication of the results. Generally, access to data of statutory health insurance funds for research purposes is possible only under the conditions defined in German Social Law (SGB V § 287). Requests for data access can be sent as a formal proposal specifying the recipient and purpose of the data transfer to the appropriate data protection agency. Access to the data used in this study can only be provided to external parties under the conditions of the cooperation contract of this research project and after written approval by the sickness fund. For assistance in obtaining access to the data, please contact wido@wido.bv.aok.de.

## Abbreviations

ACSC: Outpatient-care sensitive conditions
CI: Confidence intervals
ENT: Ear, nose, and throat
GOP: Gebührenordnungsposition
GP: Outpatient general practitioner
ICD-10-GM: International Classification of Diseases– 10. Revision– German Modification
ICU: Intensive care unit
IQR: Interquartile range
PSS: Post-Sepsis-Syndrome
SD: Standard deviation
WIdO: AOK Research Institute
