# Supplementary material for: Transitional care after hospitalization for sepsis in Germany– results from the population-based AVENIR cohort study
Source: Infection. 2025 Jul 8;53(6):2533–42. doi: 10.1007/s15010-025-02589-7 (PMC12675533; doi:10.1007/s15010-025-02589-7)
Supplement: Supplementary file 1 — Supplementary Material 1 [file 15010_2025_2589_MOESM1_ESM.docx]

**Supplementary material**

**Transitional care after hospitalization for sepsis in Germany – results from the population-based AVENIR cohort study**

Thomas Ruhnke^1^, Josephine Storch^2^, Dr. rer. pol./habil. med. Antje Freytag^2^, Dr. phil. Norman Rose^3,4^, Dr. med. Aurelia Kimmig^3^, Patrik Dröge^1^, Lisa Wedekind^5^, Christian Günster^1^, Ludwig Goldhahn^6^, apl. Prof. Dr. rer. biol. hum. habil. Enno Swart^6^, Prof. Dr. med. Mathias W. Pletz^3,4^, Prof. Dr. med. Konrad Reinhart^7^, Univ.-Prof. Dr. med. Peter Schlattmann^5^, Dr. med. Carolin Fleischmann-Struzek^3,4^

1 AOK Research Institute (WIdO), Berlin, Germany

2 Institute of General Practice and Family Medicine, Jena University Hospital, Jena, Germany

3 Institute of Infectious Diseases and Infection Control, Jena University Hospital, Jena, Germany

4 Center for Sepsis Control and Care, Jena University Hospital, Jena, Germany

5 Institute of Medical Statistics, Computer and Data Sciences, Jena University Hospital, Jena, Germany

6 Institute of Social Medicine and Health Systems Research (ISMHSR), Otto-von-Guericke-University Magdeburg, Magdeburg, Germany

7 Sepsis Foundation, Berlin, Germany

**A - Sepsis definitions**

**Table S1** Sepsis definition criteria in health claims data

| Year(s) | Sepsis and Infection ICD-10-GM Codes | Organ dysfunction ICD-10-GM or OPS Codes |
| --- | --- | --- |
| 2016-2019 | R57.2 (septic shock) OR |  |
|  | R65.1 (severe sepsis) OR |  |
|  | At least one explicit sepsis code (A02.1, A20.0, A20.7, A21.7, A22.7, A24.1, A26.7, A28.2, A32.7, A39.1, A39.2, A39.3, A39.4, A40, A41, A42.7, A48.3, A49.9, A54.8, B00.7, B37.6, B37.7, B49, O75.3, O85, T80.2, T81.4, T88.0) | AND at least one ICD-10-GM code for organ dysfunction (J80.0, J95.1, J95.2,  J96.0, J96.9, R09.2, D65, D69.57, D69.58, D69.59, D69.6, R57.8, E86, I95.9, I46.0, I46.9, R57.2, K72.0, K72.9, R17, N17.02, N17.03, N17.12 , N17.13, N17.22, N17.23, N17.82, N17.83, N17.92, N17.93, F05,  G94.32*, K72.72!, K72.73!, K72.74!, R40, G93.4) OR OPS code for organ dysfunction (8-701,* 8-704,* 8-706,* 5-311,* 5-312,* 8-800.6, 8-800.d, 8-800.f, 8-800.g, 8-800.h, 8-800.j, 8-800.k, 8-800.m, 8-800.n, 8-810.j, 8-810.x, 8 812.5, 8-812.6, 8-812.8, 8-771, 8-779, 8-852, 8-858, 8-821.2, 8-853.1, 8-853.3, 8-853.4, 8-853.5, 8-853.6, 8-853.7, 8-853.8, 8-853.x, 8-853.y, 8-854.2, 8-854.3, 8-854.4, 8-854.5, 8-854.6, 8-854.7, 8-854.8, 8-854.x, 8-854.y, 8-855.1, 8-855.3, 8-855.4, 8-855.5, 8-855.6, 8-855.7, 8-855.8, 8-855.x, 8-855.y, 8-856 , 8-85a.0, 8-85a.1) |
| 2020 | R57.2 (septic shock) OR |  |
|  | At least one explicit sepsis code (see above) | AND R65.1 (SIRS with organ dysfunction) OR |
|  | At least one explicit sepsis code (see above) | AND at least one ICD-10-GM code for organ dysfunction (see above) OR OPS code for organ dysfunction (see above)  OR |
|  | J09 OR J10 (laboratory confirmed Influenza) OR U07.1! (laboratory confirmed SARS-CoV2-infection) | AND at least one ICD-10-GM OR OPS code for organ dysfunction (see above) |

* codes were included in the definition if at least one hour of mechanical ventilation was documented in the course of the hospitalization

Abbreviations: ICD-10: International Classification of Diseases Version 10, German Modification, OPS: German Procedure Classification, Operationen- und Prozedurenschlüssel

**B - Subgroups definitions**

Septic shock at index hospitalization

ICD-10-GM Codes: R57.2 - Septic shock

ICU complex-treated sepsis at index hospitalization

Operation and Procedural Codes (OPS) Codes: 8-980 - Intensive care complex treatment, 8-98f - Costly intensive care complex treatment (basic procedure), 8-98d - Intensive care complex treatment in childhood (basic procedure)

**C – Capitation fees and physician group of the lifelong physician number for physician categories**

The following table shows the capitation fees and physician group of the lifelong physician number for categorizing the physician categories. For collective billing cases, the nationwide capitation fees were used to categorize the physician specialties. In the case of outpatient billing cases with several capitation fees, the physician group of the lifelong physician number was used in addition. Furthermore, there were regional capitation fees used. For selective billing cases, the physician group of the lifelong physician number was used for categorization.

**Table S2** capitation fees and physician group of the lifelong physician number in health claims data

|  | capitation fees | physician group of the lifelong physician number |
| --- | --- | --- |
| general practitioner (GP) | 03001,03002,03003,03004,03005,03011,03012,03013,03014,03015,03030 | 1,2,3 |
| ENT specialist | 09210,09211,09212 | 19,20 |
| cardiologist | 13540,13541,13542 | 28 |
| nephrologist | 13590,13591,13592 | 29 |
| neurologist | 16210,16211,16212 | 51,52,53 |
| pneumologist | 13640,13641,13642 | 30 |
| psychiatrist | 21210,21211,21212,21213,21214,21215 | 58,59 |
| psychologist | 23210,23211,23212,23214 | 61,68,69 |
| physician with specialization in psychosomatics | 22210,22211,22212 | 60 |
| pain treatment, pain therapy | 30700 | - |
| urologist | 26210,26211,26212 | 67 |
| emergency | 01205,01207,01210,01212,01214,01216,01218,01418 | - |

**D – Outcomes**

Outpatient emergency service of the statutory health insurance physicians

Billing of an outpatient emergency capitation fee at a facility outside a hospital

Emergency departments in hospitals (without inpatient admission)

Billing of an outpatient emergency capitation fee in a hospital

Hospital stay with emergency admission

Inpatient stay with ´07´ on the 3rd and 4th position of the admission status

**E – Results**

**Table S3** Health care utilization in 2016-2019 in the 90 days post-discharge, index survivors by age

| **Outcome** | **< 40 y** | | **40-64 y** | | **65-79 y** | | **≥ 80 y** | |
| --- | --- | --- | --- | --- | --- | --- | --- | --- |
|  | **N = 7,453** | **95% CI***^1^* | **N = 55,455** | **95% CI***^1^* | **N = 91,151** | **95% CI***^1^* | **N = 80,815** | **95% CI***^1^* |
| At least one outpatient physician contact, n (%) | 7,041 (94.5%) | [94.0%; 95.0%] | 53,417 (96.3%) | [96.2%; 96.5%] | 88,291 (96.9%) | [96.7%; 97.0%] | 78,081 (96.6%) | [96.5%; 96.7%] |
| Number of outpatient physician contacts, mean (SD); median (Q1; Q3) | 6.6 (9.4); 5 (2; 8) |  | 7.1 (7.1); 6 (3; 9) |  | 7.0 (5.1); 6 (4; 9) |  | 6.6 (4.8); 6 (3; 9) |  |
| At least one outpatient general practitioner contact, n (%) | 6,431 (86.3%) | [85.5%; 87.1%] | 51,585 (93.0%) | [92.8%; 93.2%] | 86,522 (94.9%) | [94.8%; 95.1%] | 77,067 (95.4%) | [95.2%; 95.5%] |
| Number of outpatient general practitioner contact, mean (SD); median (Q1; Q3) | 4.3 (7.2); 3 (1; 5) |  | 4.9 (5.1); 4 (2; 6) |  | 5.1 (4.0); 4 (3; 7) |  | 5.3 (4.1); 4 (3; 7) |  |
| At least one outpatient specialist contact, n (%) | 4,736 (63.5%) | [62.5%; 64.6%] | 35,008 (63.1%) | [62.7%; 63.5%] | 56,556 (62.0%) | [61.7%; 62.4%] | 41,832 (51.8%) | [51.4%; 52.1%] |
| Number of outpatient specialist contact, mean (SD); median (Q1; Q3), in detail: | 2.3 (5.6); 1 (0; 3) |  | 2.2 (4.8); 1 (0; 3) |  | 1.9 (3.0); 1 (0; 3) |  | 1.3 (2.2); 1 (0; 2) |  |
| … ENT specialist | 513 (6.9%) | [6.3%; 7.5%] | 4,123 (7.4%) | [7.2%; 7.7%] | 7,002 (7.7%) | [7.5%; 7.9%] | 6,291 (7.8%) | [7.6%; 8.0%] |
| … cardiologist | 266 (3.6%) | [3.1%; 4.0%] | 3,237 (5.8%) | [5.6%; 6.0%] | 6,496 (7.1%) | [7.0%; 7.3%] | 3,871 (4.8%) | [4.6%; 4.9%] |
| … nephrologist | 503 (6.7%) | [6.2%; 7.3%] | 4,450 (8.0%) | [7.8%; 8.3%] | 6,921 (7.6%) | [7.4%; 7.8%] | 3,610 (4.5%) | [4.3%; 4.6%] |
| … neurologist | 313 (4.2%) | [3.7%; 4.7%] | 2,818 (5.1%) | [4.9%; 5.3%] | 4,338 (4.8%) | [4.6%; 4.9%] | 3,424 (4.2%) | [4.1%; 4.4%] |
| … pneumologist | 239 (3.2%) | [2.8%; 3.6%] | 3,064 (5.5%) | [5.3%; 5.7%] | 4,500 (4.9%) | [4.8%; 5.1%] | 1,647 (2.0%) | [1.9%; 2.1%] |
| … psychiatrist | 524 (7.0%) | [6.5%; 7.6%] | 4,844 (8.7%) | [8.5%; 9.0%] | 6,714 (7.4%) | [7.2%; 7.5%] | 6,708 (8.3%) | [8.1%; 8.5%] |
| … psychologist | 164 (2.2%) | [1.9%; 2.5%] | 542 (1.0%) | [0.9%; 1.1%] | 110 (0.12%) | [0.10%; 0.14%] | 19 (0.02%) | [0.01%; 0.03%] |
| … physician with specialization in psychosomatics | 13 (0.17%) | [0.08%; 0.27%] | 57 (0.10%) | [0.08%; 0.13%] | 15 (0.02%) | [0.01%; 0.02%] | <10 |  |
| … pain treatment, pain therapy | 51 (0.68%) | [0.50%; 0.87%] | 567 (1.02%) | [0.94%; 1.11%] | 785 (0.86%) | [0.80%; 0.92%] | 437 (0.54%) | [0.49%; 0.59%] |
| … urologist | 581 (7.8%) | [7.2%; 8.4%] | 6,859 (12.4%) | [12.1%; 12.6%] | 16,969 (18.6%) | [18.4%; 18.9%] | 13,810 (17.1%) | [16.8%; 17.3%] |
| At least one inpatient hospital admission, n (%) | 3,373 (45.3%) | [44.1%; 46.4%] | 27,766 (50.1%) | [49.7%; 50.5%] | 46,044 (50.5%) | [50.2%; 50.8%] | 34,864 (43.1%) | [42.8%; 43.5%] |
| Number of inpatient hospital admissions, mean (SD); median (Q1; Q3) | 1.0 (1.7); 0 (0; 1) |  | 0.9 (1.3); 1 (0; 1) |  | 0.9 (1.2); 1 (0; 1) |  | 0.6 (0.9); 0 (0; 1) |  |
| At least one outpatient hospital treatment, n (%) | 1,893 (25.4%) | [24.4%; 26.4%] | 9,002 (16.2%) | [15.9%; 16.5%] | 8,968 (9.8%) | [9.6%; 10.0%] | 4,068 (5.0%) | [4.9%; 5.2%] |
| Number of outpatient hospital treatments, mean (SD); median (Q1; Q3) | 0.4 (0.8); 0 (0; 1) |  | 0.2 (0.6); 0 (0; 0) |  | 0.1 (0.5); 0 (0; 0) |  | 0.1 (0.3); 0 (0; 0) |  |
| At least one inpatient rehabilitation, n (%) | 610 (8.2%) | [7.6%; 8.8%] | 7,122 (12.8%) | [12.6%; 13.1%] | 14,202 (15.6%) | [15.3%; 15.8%] | 6,787 (8.4%) | [8.2%; 8.6%] |
| Number of inpatient rehabilitations, mean (SD); median (Q1; Q3) | 0.1 (0.3); 0 (0; 0) |  | 0.1 (0.4); 0 (0; 0) |  | 0.2 (0.4); 0 (0; 0) |  | 0.1 (0.3); 0 (0; 0) |  |
| At least one emergency treatment, n (%) | 2,778 (37.3%) | [36.2%; 38.4%] | 22,028 (39.7%) | [39.3%; 40.1%] | 39,743 (43.6%) | [43.3%; 43.9%] | 35,899 (44.4%) | [44.1%; 44.8%] |
| Number of emergency treatments, mean (SD); median (Q1; Q3) | 0.7 (1.4); 0 (0; 1) |  | 0.7 (1.3); 0 (0; 1) |  | 0.8 (1.2); 0 (0; 1) |  | 0.8 (1.2); 0 (0; 1) |  |
| At least one treatment by outpatient emergency service of the statutory health insurance physicians, n (%) | 620 (8.3%) | [7.7%; 8.9%] | 5,272 (9.5%) | [9.3%; 9.8%] | 11.323 (12.4%) | [12.2%; 12.6%] | 12,925 (16.0%) | [15.7%; 16.2%] |
| At least one treatment in emergency departments in hospitals (without inpatient admission), n (%) | 1,017 (13.6%) | [12.9%; 14.4%] | 5,919 (10.7%) | [10.4%; 10.9%] | 9,035 (9.9%) | [9.7%; 10.1%] | 8,145 (10.1%) | [9.9%; 10.3%] |
| At least one hospital stay with emergency admission, n (%) | 1,935 (26.0%) | [25.0%; 27.0%] | 17,043 (30.7%) | [30.3%; 31.1%] | 31,280 (34.3%) | [34.0%; 34.6%] | 26,401 (32.7%) | [32.3%; 33.0%] |
| *^1^* CI = Confidence Interval | | | | | | | | |

**Table S4** Health care utilization in 2016-2019 in the 90 days post-discharge, index survivors by nursing care dependency before index event

| **Outcome** | **No nursing care dependency** | | **Care level ≥ 2** | | **Nursing home** | |
| --- | --- | --- | --- | --- | --- | --- |
|  | **N = 133,133** | **95% CI***^1^* | **N = 71,531** | **95% CI***^1^* | **N = 30,210** | **95% CI***^1^* |
| At least one outpatient physician contact, n (%) | 128,861 (96.8%) | [96.7%; 96.9%] | 68,921 (96.4%) | [96.2%; 96.5%] | 29,048 (96.2%) | [95.9%; 96.4%] |
| Number of outpatient physician contacts, mean (SD); median (Q1; Q3) | 6.9 (6.1); 6 (4; 9) |  | 6.5 (5.1); 6 (3; 9) |  | 7.5 (5.4); 7 (4; 10) |  |
| At least one outpatient general practitioner contact, n (%) | 125,537 (94.3%) | [94.2%; 94.4%] | 67,429 (94.3%) | [94.1%; 94.4%] | 28,639 (94.8%) | [94.5%; 95.1%] |
| Number of outpatient general practitioner contact, mean (SD); median (Q1; Q3) | 4.9 (4.5); 4 (2; 6) |  | 5.1 (4.2); 4 (2; 7) |  | 6.0 (4.7); 5 (3; 8) |  |
| At least one outpatient specialist contact, n (%) | 82,911 (62.3%) | [62.0%; 62.5%] | 37,889 (53.0%) | [52.6%; 53.3%] | 17,332 (57.4%) | [56.8%; 57.9%] |
| Number of outpatient specialist contact, mean (SD); median (Q1; Q3), in detail: | 2.0 (4.0); 1 (0; 3) |  | 1.5 (2.7); 1 (0; 2) |  | 1.5 (2.2); 1 (0; 2) |  |
| … ENT specialist | 10,693 (8.0%) | [7.9%; 8.2%] | 4,579 (6.4%) | [6.2%; 6.6%] | 2,657 (8.8%) | [8.5%; 9.1%] |
| … cardiologist | 10,309 (7.7%) | [7.6%; 7.9%] | 3,124 (4.4%) | [4.2%; 4.5%] | 437 (1.4%) | [1.3%; 1.6%] |
| … nephrologist | 9,124 (6.9%) | [6.7%; 7.0%] | 5,418 (7.6%) | [7.4%; 7.8%] | 942 (3.1%) | [2.9%; 3.3%] |
| … neurologist | 4,919 (3.7%) | [3.6%; 3.8%] | 3,243 (4.5%) | [4.4%; 4.7%] | 2,731 (9.0%) | [8.7%; 9.4%] |
| … pneumologist | 6,840 (5.1%) | [5.0%; 5.3%] | 2,352 (3.3%) | [3.2%; 3.4%] | 258 (0.85%) | [0.75%; 0.96%] |
| … psychiatrist | 6,642 (5.0%) | [4.9%; 5.1%] | 5,409 (7.6%) | [7.4%; 7.8%] | 6,739 (22.3%) | [21.8%; 22.8%] |
| … psychologist | 708 (0.53%) | [0.49%; 0.57%] | 120 (0.17%) | [0.14%; 0.20%] | <10 |  |
| … physician with specialization in psychosomatics | 81 (0.06%) | [0.05%; 0.07%] | <10 |  | < 10 |  |
| … pain treatment, pain therapy | 985 (0.74%) | [0.69%; 0.79%] | 733 (1.02%) | [0.95%; 1.10%] | 122 (0.40%) | [0.33%; 0.48%] |
| … urologist | 19,789 (14.9%) | [14.7%; 15.1%] | 11,690 (16.3%) | [16.1%; 16.6%] | 6,740 (22.3%) | [21.8%; 22.8%] |
| At least one inpatient hospital admission, n (%) | 63,396 (47.6%) | [47.4%; 47.9%] | 35,950 (50.3%) | [49.9%; 50.6%] | 12,701 (42.0%) | [41.5%; 42.6%] |
| Number of inpatient hospital admissions, mean (SD); median (Q1; Q3) | 0.8 (1.2); 0 (0; 1) |  | 0.8 (1.1); 1 (0; 1) |  | 0.6 (0.9); 0 (0; 1) |  |
| At least one outpatient hospital treatment, n (%) | 15,585 (11.7%) | [11.5%; 11.9%] | 5,536 (7.7%) | [7.5%; 7.9%] | 2,810 (9.3%) | [9.0%; 9.6%] |
| Number of outpatient hospital treatments, mean (SD); median (Q1; Q3) | 0.2 (0.5); 0 (0; 0) |  | 0.1 (0.4); 0 (0; 0) |  | 0.1 (0.4); 0 (0; 0) |  |
| At least one inpatient rehabilitation, n (%) | 22,921 (17.2%) | [17.0%; 17.4%] | 5,431 (7.6%) | [7.4%; 7.8%] | 369 (1.2%) | [1.1%; 1.3%] |
| Number of inpatient rehabilitations, mean (SD); median (Q1; Q3) | 0.2 (0.4); 0 (0; 0) |  | 0.1 (0.3); 0 (0; 0) |  | 0.0 (0.1); 0 (0; 0) |  |
| At least one emergency treatment, n (%) | 50,743 (38.1%) | [37.9%; 38.4%] | 34,728 (48.5%) | [48.2%; 48.9%] | 14,977 (49.6%) | [49.0%; 50.1%] |
| Number of emergency treatments, mean (SD); median (Q1; Q3) | 0.7 (1.1); 0 (0; 1) |  | 0.9 (1.3); 0 (0; 1) |  | 0.9 (1.3); 0 (0; 1) |  |
| At least one treatment by outpatient emergency service of the statutory health insurance physicians, n (%) | 12,029 (9.0%) | [8.9%; 9.2%] | 11,302 (15.8%) | [15.5%; 16.1%] | 6,809 (22.5%) | [22.1%; 23.0%] |
| At least one treatment in emergency departments in hospitals (without inpatient admission), n (%) | 12,436 (9.3%) | [9.2%; 9.5%] | 7,820 (10.9%) | [10.7%; 11.2%] | 3,860 (12.8%) | [12.4%; 13.2%] |
| At least one hospital stay with emergency admission, n (%) | 39,446 (29.6%) | [29.4%; 29.9%] | 27,039 (37.8%) | [37.4%; 38.2%] | 10,174 (33.7%) | [33.1%; 34.2%] |
| *^1^* CI = Confidence Interval | | | | | | |

**Table S5** Health care utilization in 2016-2019 in the 90 days post-discharge, index survivors by intensive care unit (ICU) complex treatment during index event

| **Outcome** | **None ICU-treated** | | **ICU-treated** | |
| --- | --- | --- | --- | --- |
|  | **N = 147,878** | **95% CI***^1^* | **N = 86,996** | **95% CI***^1^* |
| At least one outpatient physician contact, n (%) | 143,169 (96.8%) | [96.7%; 96.9%] | 83,661 (96.2%) | [96.0%; 96.3%] |
| Number of outpatient physician contacts, mean (SD); median (Q1; Q3) | 6.7 (5.4); 6 (3; 9) |  | 7.1 (6.2); 6 (4; 9) |  |
| At least one outpatient general practitioner contact, n (%) | 139,699 (94.5%) | [94.4%; 94.6%] | 81,906 (94.1%) | [94.0%; 94.3%] |
| Number of outpatient general practitioner contact, mean (SD); median (Q1; Q3) | 5.0 (4.3); 4 (2; 6) |  | 5.3 (4.7); 4 (3; 7) |  |
| At least one outpatient specialist contact, n (%) | 87,050 (58.9%) | [58.6%; 59.1%] | 51,082 (58.7%) | [58.4%; 59.0%] |
| Number of outpatient specialist contact, mean (SD); median (Q1; Q3), in detail: | 1.7 (3.2); 1 (0; 2) |  | 1.8 (3.8); 1 (0; 2) |  |
| … ENT specialist | 10,816 (7.3%) | [7.2%; 7.4%] | 7,113 (8.2%) | [8.0%; 8.4%] |
| … cardiologist | 7,950 (5.4%) | [5.3%; 5.5%] | 5,920 (6.8%) | [6.6%; 7.0%] |
| … nephrologist | 9,431 (6.4%) | [6.3%; 6.5%] | 6,053 (7.0%) | [6.8%; 7.1%] |
| … neurologist | 6,831 (4.6%) | [4.5%; 4.7%] | 4,062 (4.7%) | [4.5%; 4.8%] |
| … pneumologist | 5,330 (3.6%) | [3.5%; 3.7%] | 4,120 (4.7%) | [4.6%; 4.9%] |
| … psychiatrist | 12,173 (8.2%) | [8.1%; 8.4%] | 6,617 (7.6%) | [7.4%; 7.8%] |
| … psychologist | 432 (0.29%) | [0.26%; 0.32%] | 403 (0.46%) | [0.42%; 0.51%] |
| … physician with specialization in psychosomatics | 53 (0.04%) | [0.03%; 0.05%] | 39 (0.04%) | [0.03%; 0.06%] |
| … pain treatment, pain therapy | 1,122 (0.76%) | [0.71%; 0.80%] | 718 (0.83%) | [0.77%; 0.89%] |
| … urologist | 25,352 (17.1%) | [17.0%; 17.3%] | 12,867 (14.8%) | [14.6%; 15.0%] |
| At least one inpatient hospital admission, n (%) | 68,345 (46.2%) | [46.0%; 46.5%] | 43,702 (50.2%) | [49.9%; 50.6%] |
| Number of inpatient hospital admissions, mean (SD); median (Q1; Q3) | 0.8 (1.1); 0 (0; 1) |  | 0.8 (1.1); 1 (0; 1) |  |
| At least one outpatient hospital treatment, n (%) | 14,195 (9.6%) | [9.4%; 9.7%] | 9,736 (11.2%) | [11.0%; 11.4%] |
| Number of outpatient hospital treatments, mean (SD); median (Q1; Q3) | 0.1 (0.5); 0 (0; 0) |  | 0.2 (0.5); 0 (0; 0) |  |
| At least one inpatient rehabilitation, n (%) | 9,784 (6.6%) | [6.5%; 6.7%] | 18,937 (21.8%) | [21.5%; 22.0%] |
| Number of inpatient rehabilitations, mean (SD); median (Q1; Q3) | 0.1 (0.3); 0 (0; 0) |  | 0.2 (0.5); 0 (0; 0) |  |
| At least one emergency treatment, n (%) | 61,968 (41.9%) | [41.7%; 42.2%] | 38,480 (44.2%) | [43.9%; 44.6%] |
| Number of emergency treatments, mean (SD); median (Q1; Q3) | 0.7 (1.2); 0 (0; 1) |  | 0.8 (1.3); 0 (0; 1) |  |
| At least one treatment by outpatient emergency service of the statutory health insurance physicians, n (%) | 19,146 (12.9%) | [12.8%; 13.1%] | 10,994 (12.6%) | [12.4%; 12.9%] |
| At least one treatment in emergency departments in hospitals (without inpatient admission), n (%) | 14,461 (9.8%) | [9.6%; 9.9%] | 9,655 (11.1%) | [10.9%; 11.3%] |
| At least one hospital stay with emergency admission, n (%) | 47,017 (31.8%) | [31.6%; 32.0%] | 29,642 (34.1%) | [33.8%; 34.4%] |
| *^1^* CI = Confidence Interval | | | | |

**Fig. S1** First health service provider contacts in the 90 days post-discharge 2016-2019 by age

**Fig. S1a** First health service provider contacts in the 90 days post-discharge 2016-2019 by age < 40 y


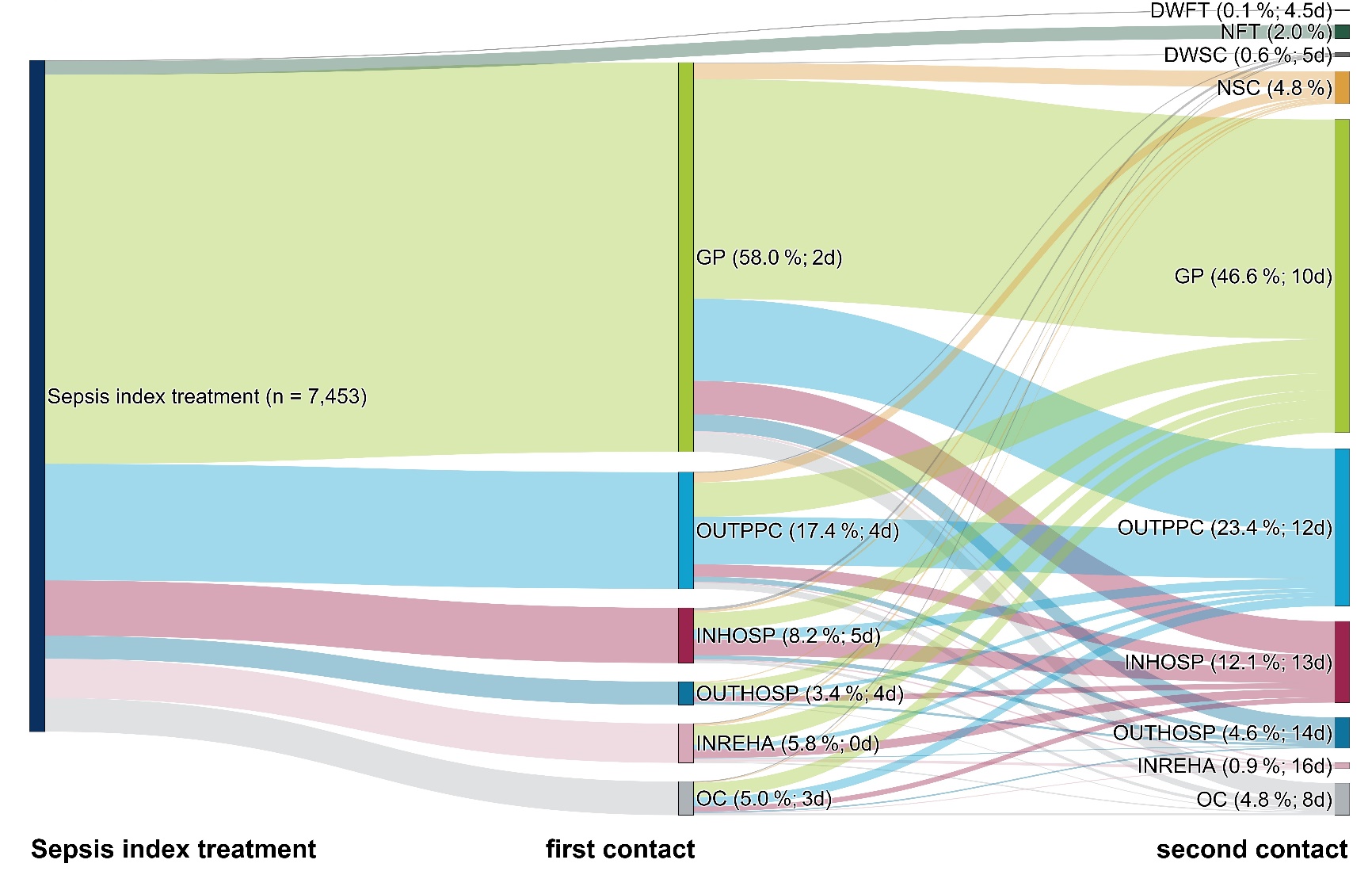


Legend for first and second contact: Category (proportion; Median number of days between health service provider contact and discharge from index hospitalization)

Abbreviations: DWFT= died without follow-up treatment, DWSC= died without second contact, GP = general practitioner, INHOSP=inpatient hospital admissions (including emergency admissions), INREHA = inpatient rehabilitation; NFT= no further treatment, NSC= no second contact, OC=other combinations, OUTHOSP = outpatient hospital treatment, OUTPPC=other outpatient physician-patient contact (specialists, outpatient emergency treatments)

**Fig. S1b** First health service provider contacts in the 90 days post-discharge 2016-2019 by age 40-64 y


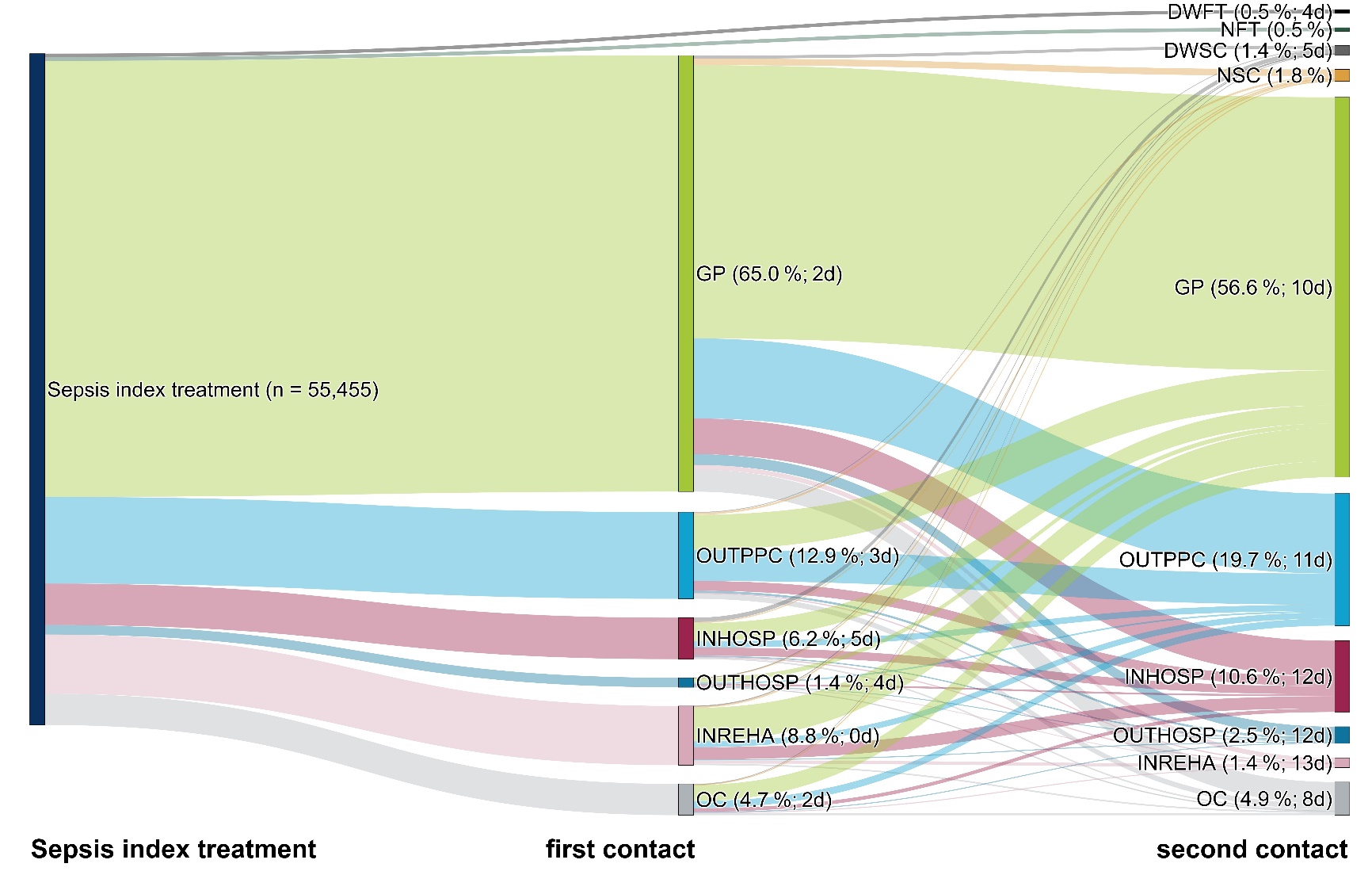


Legend for first and second contact: Category (proportion; Median number of days between health service provider contact and discharge from index hospitalization)

Abbreviations: DWFT= died without follow-up treatment, DWSC= died without second contact, GP = general practitioner, INHOSP=inpatient hospital admissions (including emergency admissions), INREHA = inpatient rehabilitation; NFT= no further treatment, NSC= no second contact, OC=other combinations, OUTHOSP = outpatient hospital treatment, OUTPPC=other outpatient physician-patient contact (specialists, outpatient emergency treatments)

**Fig. S1c** First health service provider contacts in the 90 days post-discharge 2016-2019 by age 65-79 y


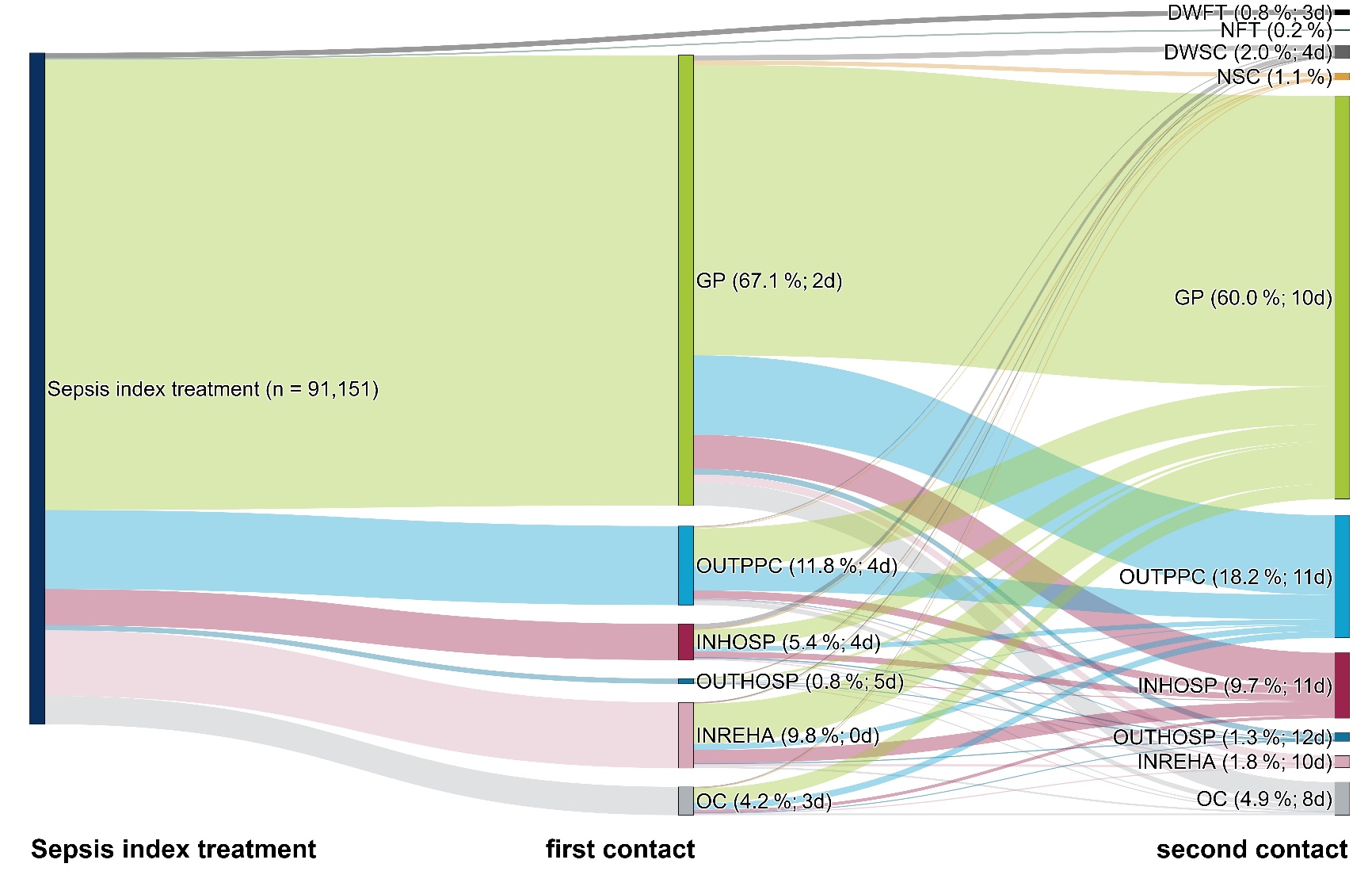


Legend for first and second contact: Category (proportion; Median number of days between health service provider contact and discharge from index hospitalization)

Abbreviations: DWFT= died without follow-up treatment, DWSC= died without second contact, GP = general practitioner, INHOSP=inpatient hospital admissions (including emergency admissions), INREHA = inpatient rehabilitation; NFT= no further treatment, NSC= no second contact, OC=other combinations, OUTHOSP = outpatient hospital treatment, OUTPPC=other outpatient physician-patient contact (specialists, outpatient emergency treatments)

**Fig. S1d** First health service provider contacts in the 90 days post-discharge 2016-2019 by age ≥ 80 y


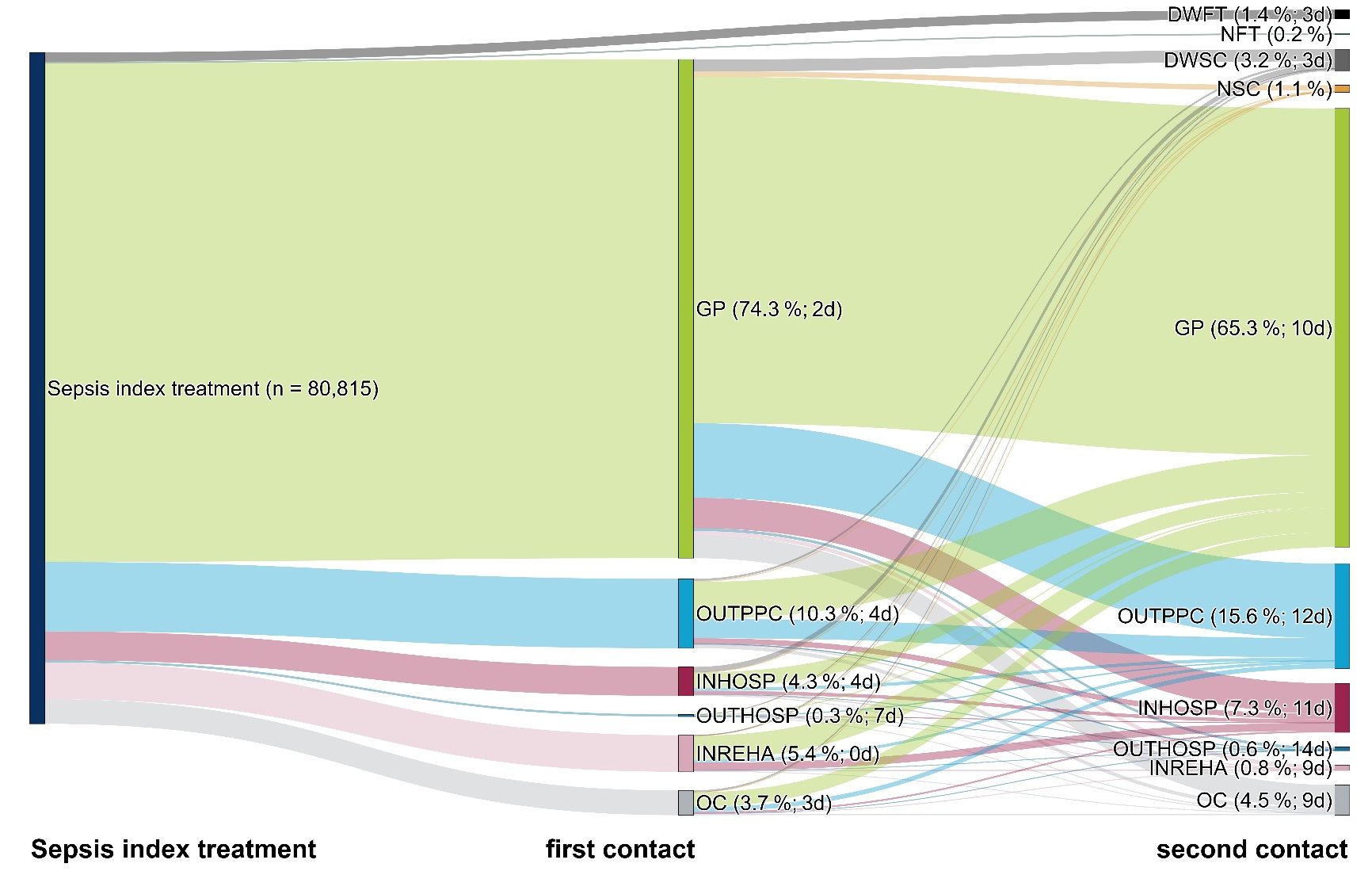


Legend for first and second contact: Category (proportion; Median number of days between health service provider contact and discharge from index hospitalization)

Abbreviations: DWFT= died without follow-up treatment, DWSC= died without second contact, GP = general practitioner, INHOSP=inpatient hospital admissions (including emergency admissions), INREHA = inpatient rehabilitation; NFT= no further treatment, NSC= no second contact, OC=other combinations, OUTHOSP = outpatient hospital treatment, OUTPPC=other outpatient physician-patient contact (specialists, outpatient emergency treatments)

**Fig. S2** First health service provider contacts in the 90 days post-discharge 2016-2019 by nursing care dependency before index event

**Fig. S2a** First health service provider contacts in the 90 days post-discharge 2016-2019 without nursing care dependency before index event


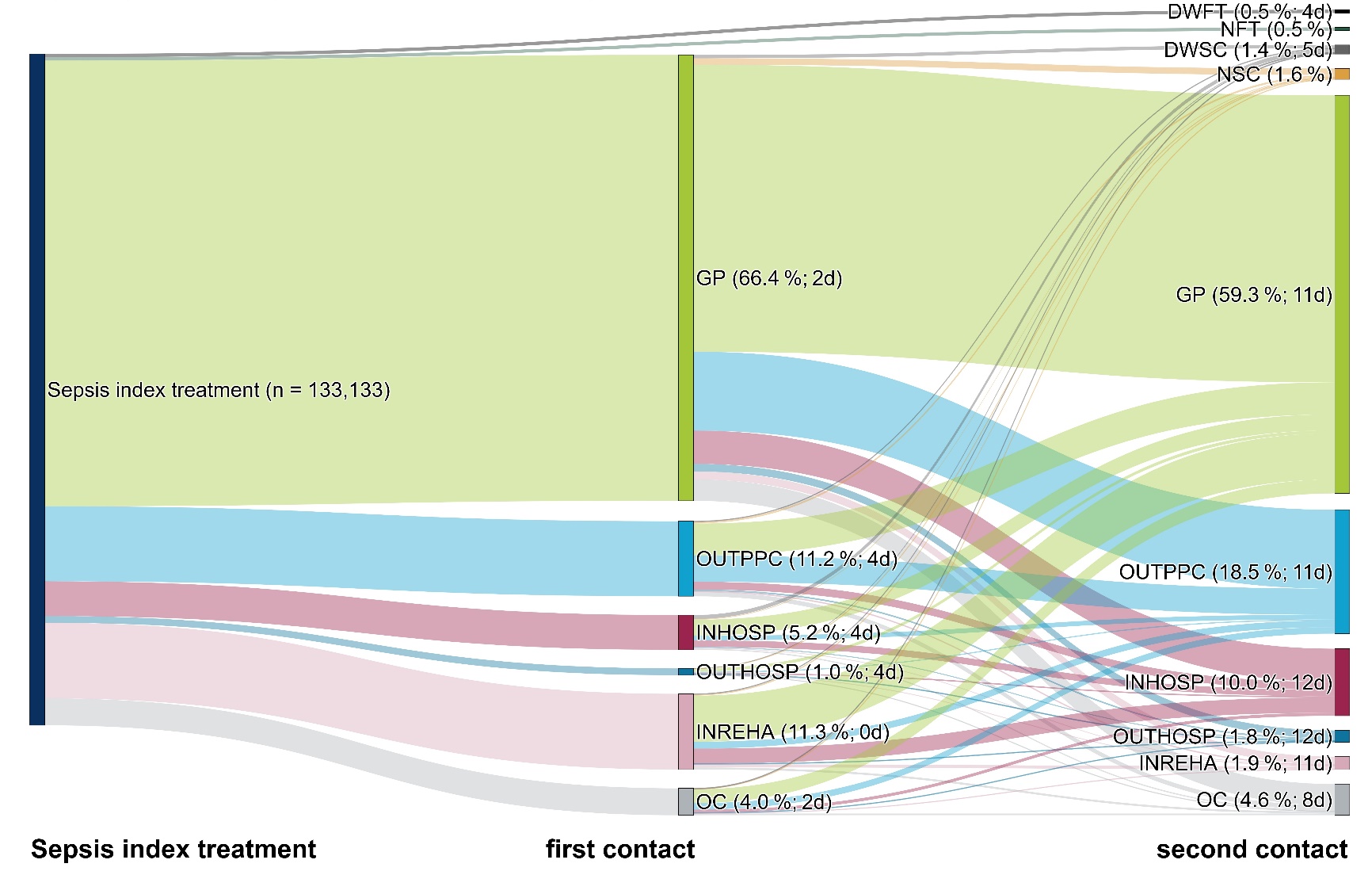


Legend for first and second contact: Category (proportion; Median number of days between health service provider contact and discharge from index hospitalization)

Abbreviations: DWFT= died without follow-up treatment, DWSC= died without second contact, GP = general practitioner, INHOSP=inpatient hospital admissions (including emergency admissions), INREHA = inpatient rehabilitation; NFT= no further treatment, NSC= no second contact, OC=other combinations, OUTHOSP = outpatient hospital treatment, OUTPPC=other outpatient physician-patient contact (specialists, outpatient emergency treatments)

**Fig. S2b** First health service provider contacts in the 90 days post-discharge 2016-2019 with care level ≥ 2 before index event


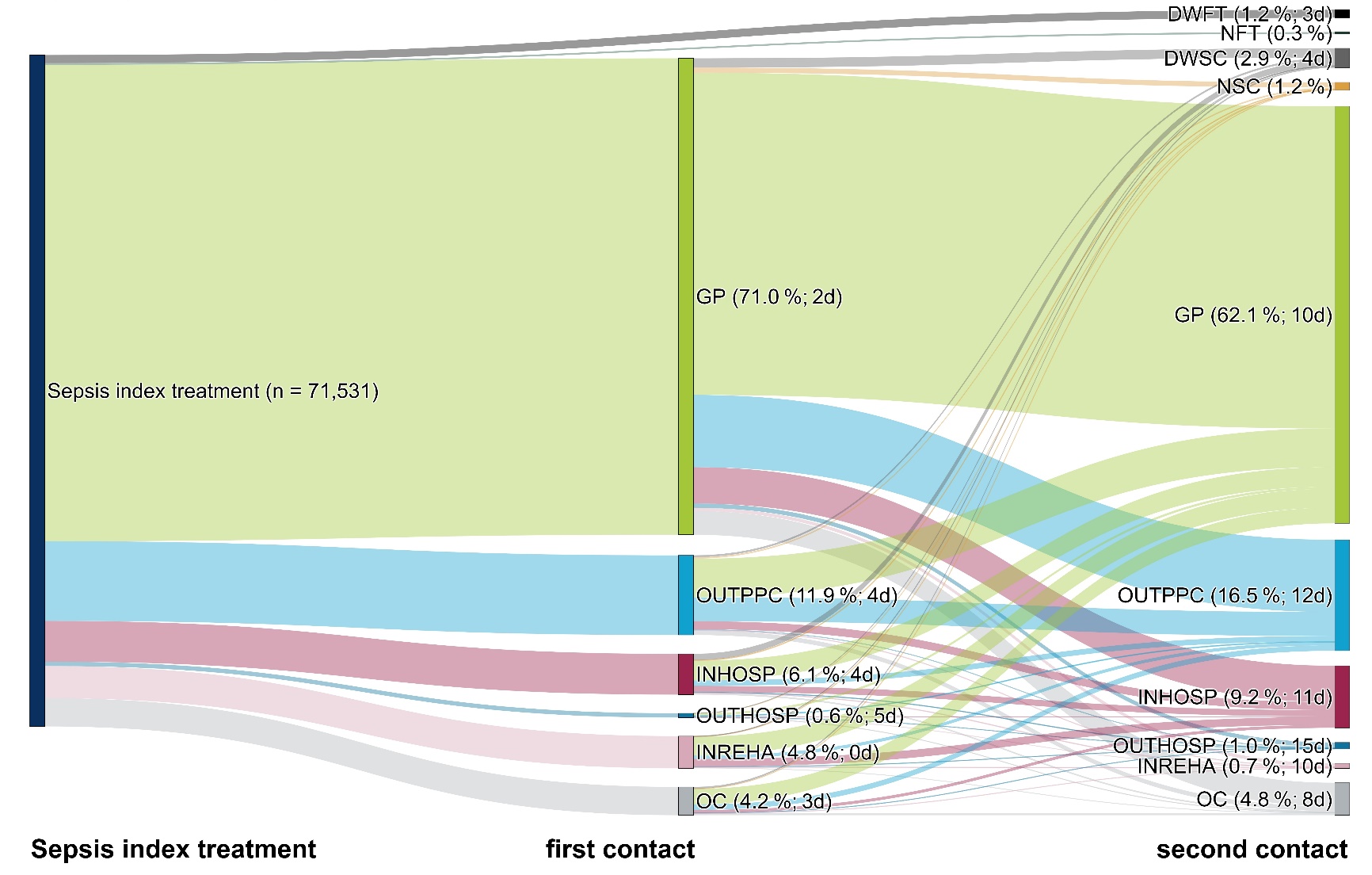


Legend for first and second contact: Category (proportion; Median number of days between health service provider contact and discharge from index hospitalization)

Abbreviations: DWFT= died without follow-up treatment, DWSC= died without second contact, GP = general practitioner, INHOSP=inpatient hospital admissions (including emergency admissions), INREHA = inpatient rehabilitation; NFT= no further treatment, NSC= no second contact, OC=other combinations, OUTHOSP = outpatient hospital treatment, OUTPPC=other outpatient physician-patient contact (specialists, outpatient emergency treatments)

**Fig. S2c** First health service provider contacts in the 90 days post-discharge 2016-2019 with nursing home before index event


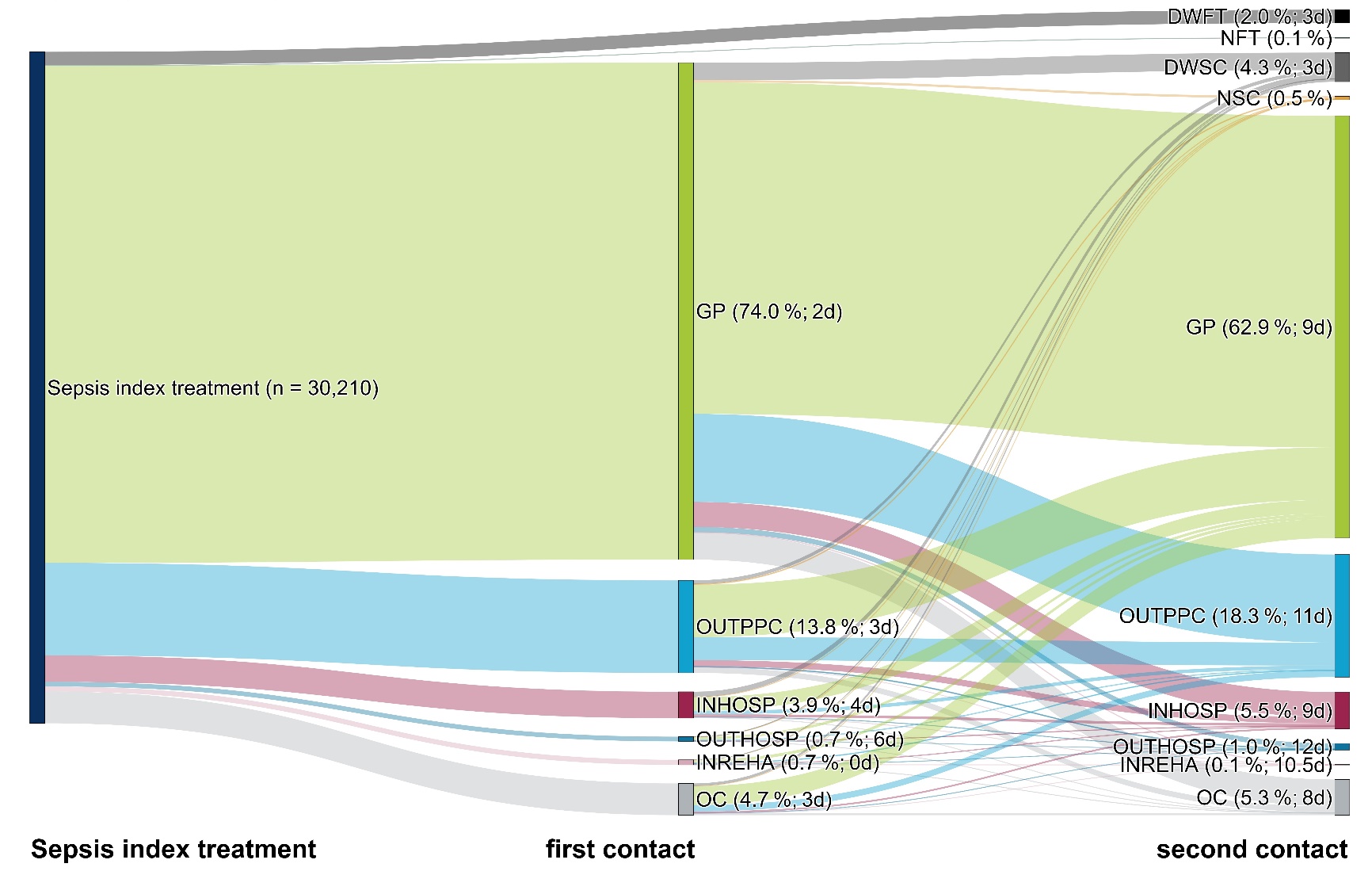


Legend for first and second contact: Category (proportion; Median number of days between health service provider contact and discharge from index hospitalization)

Abbreviations: DWFT= died without follow-up treatment, DWSC= died without second contact, GP = general practitioner, INHOSP=inpatient hospital admissions (including emergency admissions), INREHA = inpatient rehabilitation; NFT= no further treatment, NSC= no second contact, OC=other combinations, OUTHOSP = outpatient hospital treatment, OUTPPC=other outpatient physician-patient contact (specialists, outpatient emergency treatments)

**Fig. S3** First health service provider contacts in the 90 days post-discharge 2016-2019 by intensive care unit (ICU) complex treatment during index event

**Fig. S3a** First health service provider contacts in the 90 days post-discharge 2016-2019 without intensive care unit (ICU) complex treatment during index event


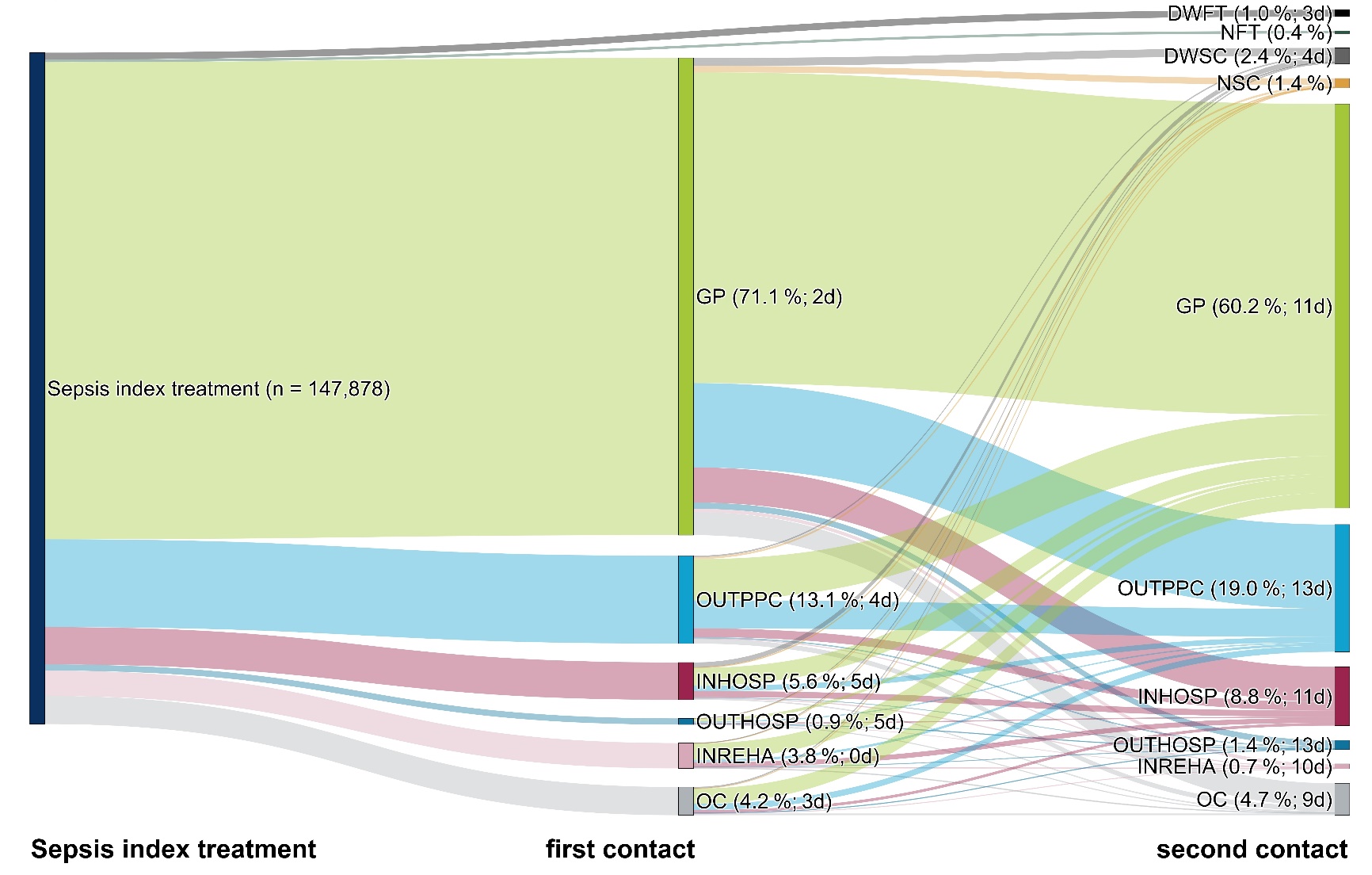


Legend for first and second contact: Category (proportion; Median number of days between provider health service contact and discharge from index hospitalization)

Abbreviations: DWFT= died without follow-up treatment, DWSC= died without second contact, GP = general practitioner, INHOSP=inpatient hospital admissions (including emergency admissions), INREHA = inpatient rehabilitation; NFT= no further treatment, NSC= no second contact, OC=other combinations, OUTHOSP = outpatient hospital treatment, OUTPPC=other outpatient physician-patient contact (specialists, outpatient emergency treatments)

**Fig. S3b** First health service provider contacts in the 90 days post-discharge 2016-2019 with intensive care unit (ICU) complex treatment during index event


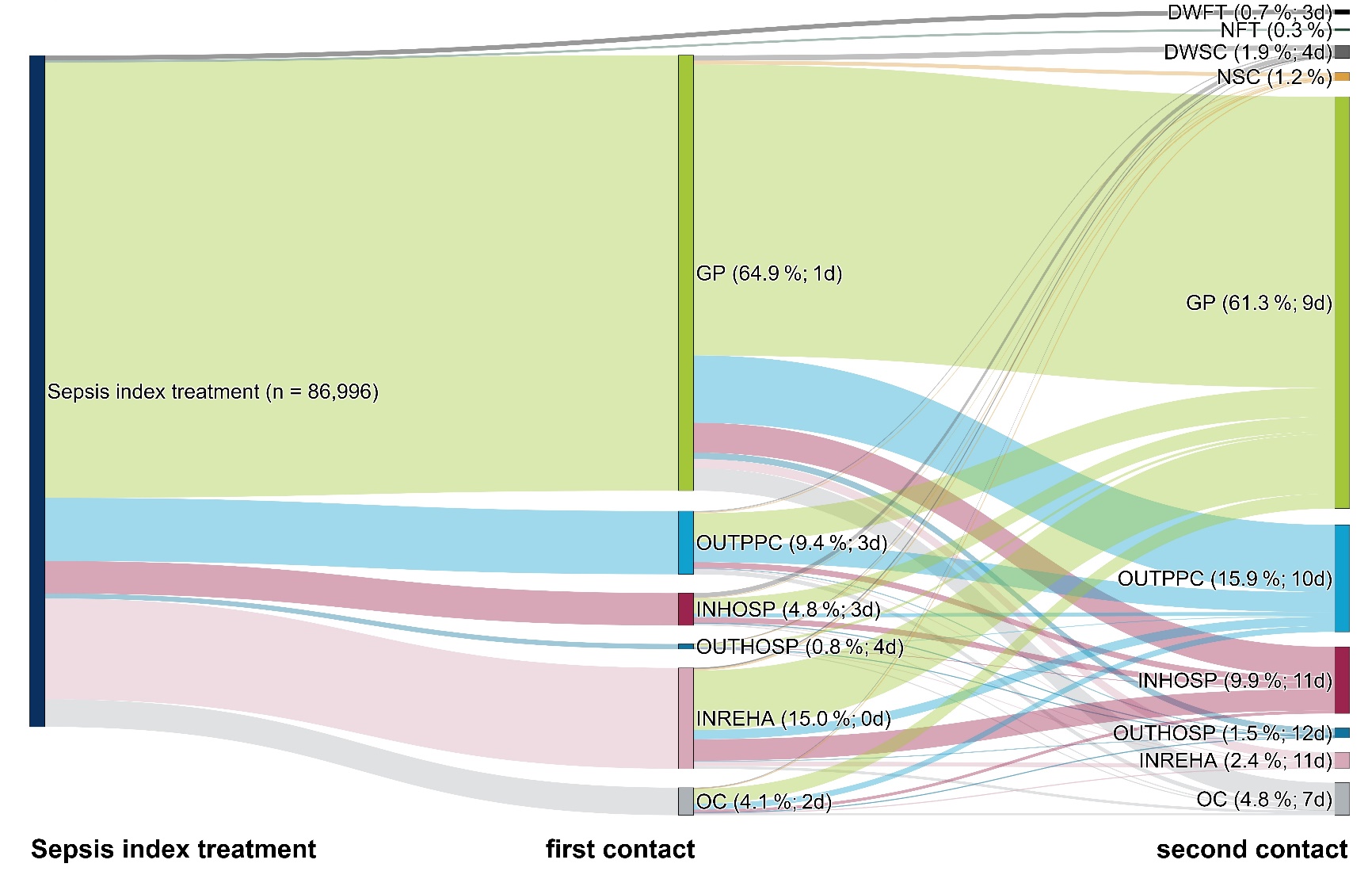


Legend for first and second contact: Category (proportion; Median number of days between health service provider contact and discharge from index hospitalization)

Abbreviations: DWFT= died without follow-up treatment, DWSC= died without second contact, GP = general practitioner, INHOSP=inpatient hospital admissions (including emergency admissions), INREHA = inpatient rehabilitation; NFT= no further treatment, NSC= no second contact, OC=other combinations, OUTHOSP = outpatient hospital treatment, OUTPPC=other outpatient physician-patient contact (specialists, outpatient emergency treatments)
